# When Challenging Art Gets Liked: Evidences for a Dual Preference Formation Process for Fluent and Non-Fluent Portraits

**DOI:** 10.1371/journal.pone.0131796

**Published:** 2015-08-26

**Authors:** Benno Belke, Helmut Leder, Claus Christian Carbon

**Affiliations:** 1 University of Vienna, Department of Basic Psychological Research and Research Methods, Faculty of Psychology, Vienna, Austria; 2 University of Bamberg, Department of General Psychology and Methodology, Bamberg, Germany; 3 Bamberg Graduate School of Affective and Cognitive Sciences, Bamberg, Germany; Northwestern University, UNITED STATES

## Abstract

Although fluency theory predominates psychological research on human aesthetics, its most severe limitation may be to explain why art that challenges or even violates easy processing can nevertheless be aesthetically rewarding. We discuss long-standing notions on art’s potential to offer mental growth opportunities and to tap into a basic epistemic predisposition that hint at a fluency counteracting aesthetic pleasure mechanism. Based on divergent strands of literature on empirical, evolutionary, and philosophical aesthetics, as well as research on disfluency, we presumed that challenging art requires deliberate reflexive processing at the level of “aboutness” in order to be experientially pleasing. Here, we probed such a cognitive mastering mechanism, achieved by iterative cycles of elaboration, as predicted by our model of aesthetic experiences. For the study, two kinds of portraits were applied, one associable to a high fluency and one to a high stimulation potential (according to results of an extensive rating study). In Experiment 1, we provided a repeated evaluation task, which revealed a distinctive preference effect for challenging portraits that was absent in the visual exposition conditions of a familiarity and a mere exposure task (Experiment 2). In a follow-up task (Experiment 3) this preference effect was observed with a novel and more encompassing pool of portraits, which corroborated its stability and robustness. In an explorative stimulus-transfer task (Experiment 4), we investigated the presumed underlying mechanism by testing whether the observed effect would generalize onto novel portraits of the same artist-specific styles. Results discounted an alternative interpretation of a perceptual adaptation effect and hinted at meaning-driven mental activity. Conjointly, findings for inexperienced viewers were indicative of an elaboration based mastering mechanism that selectively operated for mentally challenging portraits. Moreover, findings were in line with a dual-process view of human preference formation with art. Theoretical implications and boundary conditions are discussed.

## Introduction

Already Aristotle formulated in his Poetics as an elementary aesthetic rule: “The unfamiliar provides a specific pleasure resulting from admiration and astonishment of it’s source [..]; The common on the contrary is pleasurable because it is easy to process.” (II 354, cited in [1], p. 36). This antique conception still resonates in contemporary theorizations within empirical aesthetics and has remained a valuable explanation for why we are attracted to art today. From a psychological point of view, however, two complementary types of underlying mental reward-mechanisms seem to be addressed: Aesthetic pleasure derived from the confirmation and preservation as opposed to extension and growth of a viewer’s mental capabilities. In modern terms, Aristotle’s “pleasure of the common” resembles contemporary processing fluency theory, which, since the pioneering work by Jacoby and Dallas [2], has established itself as the single most influential explanation of aesthetic appreciation [3]. Fluency theory essentially posits that one particular mechanism–the phenomenal mental ease (or effort)–explains how aesthetic pleasure is derived from art [4–6]. Fluency is described as a metacognitive phenomenon [7] resulting from the constant self-assessment of the brain’s on-going mental operations and capability to deal with current processing demands [8]. In line with an affect-as-information principle [9, 10] this positively valenced fluency-cue is assumed to serve as heuristic information for the evaluation of the corresponding target stimulus. Fluency theory predicts that objects are phenomenally pleasing that are “easy on the mind” [6] and that people have a tendency to enjoy what matches their current knowledge [11] as any contribution to “enhanced” (fast, efficient, error-free) mental processing should be preferred [12–15]. Since fluency theory is rigid [16], it follows that aesthetic appreciation should arise only from what offers relative direct and effortless access to a stimulus physical identity and conveyed meaning, thus confirms a viewer’s mental processing routines and capacities.

Fluency theory is currently best revealed in the perceptual domain, where it connects a range of well-established aesthetic effects of low-level features into a coherent framework [3]. Accordingly, fluency can be embedded on a psychophysical level by facilitating perceptual encoding of a stimulus, as revealed by preference effects for symmetrical, balanced, or prototypical objects [4, 5]. This type of object-related fluency can be associated to the perception of harmony or good gestalt that have been traditionally linked to aesthetic appreciation [17]. Alternatively, fluency experiences have been proposed to result from the learning history with a stimulus [12] as revealed by effects of subjective familiarity e.g. [18], mere exposure e.g. [19–21], or visual priming e.g. [22, 23]. Moreover, fluency experiences stretch out to conceptual processing [13, 14, 24, 25] which has been investigated by means of direct priming [26, 27] and indirect priming methods [25, 28, 29]. Such cognitive fluency has been found relevant for aesthetic appreciation of paintings [30].

Noteworthy, this fluency-based view of human aesthetics ties into a growingly influential trend of neuroaesthetics to uncover so-called “aesthetic laws”, which are proposed to directly tap into basic hard-wired mental predispositions [31–34]. For instance, according to Cavanagh [35] artists often deploy simplifications that transgress standard physics (e.g. by impossible shadows) but nevertheless do not interfere with a viewer’s understanding of a depicted scene–as artists make use of a *mental* representation logic. Moreover, similarly to caricaturists, artists were proposed to exaggerate critical shape characteristics in such a “supernormal” manner that facilitates recognition of depicted objects [33] or reading of emotional body-gestures [32]. Thereby, such neuroaesthetic accounts posit a kind of structural isomorphism between principles that govern the brain’s visual routines and aesthetic principles of depiction (predicting that stronger “matches” are aesthetically favoured). Although usually not linked to fluency theory, this influential strand of research reveals a fluency-like rationale, since whether perceiving an aesthetic entity is rewarding or not is described as a matter of its “processability”.

### Limitations of fluency theory for art experiences

Despite striking empirical evidences, fluency predictions reveal some strong conceptual limitations: If there were no other qualities that could be appreciated, art would be restricted to a fixed set of “obvious” artefacts that primarily serve to maximize easy processing of the viewer and ensure its immediate non-demanding mental appropriation. While this may be true of isolated forms of *Kitsch* or decorative art this is evidently not a valid characterization of the rich fundus provided by the history of art and can hardly hold as sufficient explanation for either the conception or consumption of art today. Arguably, such strict fluency compliance may have prevented art to establish itself as a significant cultural phenomenon that stretches out to 40,000 years of existence, since there would be little left to regard artistic artefacts special. On the contrary, it is widely acknowledged that art, particularly since the upcoming of modernism, often blatantly disrupts and violates easy processing [3, 16, 36, 37]. Consequently, experimental aestheticians have for a long time debated variables that stimulate the viewing experience via novelty, conflict, or surprisingness e.g. [38–43] that were already acknowledged in Darwin’s [44] evolutionary account of aesthetics. In fact, striving for novelty can been seen as the dominant force in the development of art [45, 46]. However, fluency theory has troubles to accommodate for any mentally stimulating effects without greater theoretical stretches of its core principle [16, 43, 47].

Fluency limitations go further considering that a hallmark of art is an inherent ambiguity and semantic openness [43, 48–51]. Thus, substantial mental effort is often required to distil from a “plurality of interpretations” [52] and to fill in the gaps of what cannot be readily inferred from an incomplete or indistinct image [53, 54]. Again, this poses the problem for fluency proponents to explain why ambiguity that impedes rather than facilitates conceptual processing is aesthetically appreciable [55, 56]. Recently, attempts were made to link violations of expectancy with hedonic effects in art [17] and common objects [57]. As Ramachandran and Hirstein [33] noted “it is though an object discovered after a struggle is more pleasing than one that is instantly obvious” (p. 30). Therefore, a comprehensive psychological account of aesthetic appreciation needs to consider the positive contribution of disruptive aesthetic qualities that transcend or even conflict with a viewer’s mental processing routines and capabilities [17, 37, 43]. These characteristics may also explain why experiencing art often invokes affective states and emotions ranging from surprise, curiosity, insight, awe, and even shocks [43, 58–61], which cannot be associated to easy-to-process features [16, 62]. We propose to subsume such fluency-opposing contributors under the collective term of cognitive stimulation, which may define the level of mental challengingness (or disfluency) of an artistic relict.

### Neurocognitive benefits of challenging art

What causes the mind to appreciate challenging art? Several lines of literature unite at the idea that art offers the prospect to obtain new information and extend acquired knowledge, hence provides mental growth opportunities [16]. Aesthetic pleasure may therefore be rooted in a natural epistemic disposition to seek novel information as has been previously revealed from divergent theoretical angles on human curiosity, drive, and information seeking behaviour [63–67] (see Loewenstein [68] for a review). Berlyne, for example, in his seminal work, proposed with his notion of “epistemic curiosity” a uniquely human drive that can be understood as “the desire for knowledge that motivates individuals to learn new ideas, eliminate information-gaps, and solve intellectual problems” [69], p.1586. Recent accounts of evolutionary aesthetics parallel such claims by proposing that aesthetic experiences are marked by an “appetite for certain types of information” [61], p. 58. This idea is substantiated in a recent neuropsychological model by Biederman and Vessel [70], which identified a reward network of opioids that serve as neural substrate of the pleasure of acquiring new visual information. These notions are in line a with a recent survey on art-gallery and museum visitors in the UK that revealed as primary motivation to engage with art self-actualization and cognitive needs [71]. Challenging art seems to provide an exceedingly rich sphere for mental exploration, information seeking, and learning opportunities by introducing unique approaches to the visual representation of objects, concepts, and ideas under risk-free conditions. Thus, experiencing such art may serve what Tooby and Cosmides [72] coined a “neurocognitive adaption function”, as knowledge, even if of no immediate use, can be vicariously obtained and the cognitive system safely adapted to new experiences. Aristotle’s notion of the “pleasure of the unfamiliar” appears therefore associable with the prospect of learning–an idea that recalls a popular notion since Idealistic philosophy that art experiences would primarily serve a self-educating function for the perceiver, see [1].

### The role of cognitive mastering in art experiences

However, intense mental stimulation is also known to provoke negative affective reactions such as avoidance behaviour, disliking, and confusion [73–76]. This is reflected in countless historical events of audience outrages and critics’ dismissal of what later became canonical art, with one explanation being that high levels of arousal activate a primary avoidance system [39, 77]. In a model of aesthetic experiences [78] we proposed that the extent to which perceiving challenging art is aesthetically pleasing essentially depends on the viewer’s phenomenal state of cognitive mastering. This art-specific processing stage concerns elaboration of *represented* meaning beyond initial categorization of subject matter or style [78]. The significance of this stage is a direct consequence of art’s ontological status as a symbol or representation (and not a real-world entity) that is the foundation of influential art-philosophical theories e.g. of Husserl [79], Gombrich [48], Danto [80], Wollheim [81], or Bredekamp [41]. However, this representational status has far-reaching consequences for human cognition and corresponding preference formation that were often overlooked in experimental aesthetics. Accordingly, an artwork’s physical appearance serves as carrier and signifier of information that points beyond itself [36]. Since its manifest (psychophysical) properties are interpretanda that embody meaning this led Danto to conclude that the essence of art is the possession of “aboutness” [80]: “What we see in a painting was intended by the artist, who organised the surface in order that viewers should grasp what was meant in putting it there” [82]. Although committed to a strict reductionist view Fechner [83] reflected on the same issue by noting “stripped of all associations what remained of the Sistine Madonna would be a potpourri colour plate whose pleasure of perceiving would be surpassed by any piece of carpet” (translated by the authors, p. 118). The presumably exclusive human ability to see objects, scenes, and concepts *in* paintings (and not just paint samples) is a basic mental capacity to “accept” an image as a fictive/representational entity [1, 81]. From a cognitive evolutionary perspective, when perceiving art mental processes are switched into an “as-if” mode of symbolical cognition [1, 84, 85] that should genuinely drive perception towards abstraction of surface features, hence (in the model’s terms) cognitive mastering of”aboutness”. Cognition of art seems therefore grounded in a special type of object understanding that separates the art-world (“Kunstwelt”) from real-life situations (“Lebenswelt”). Compared to Palmeri and Gauthier’s [86] recent summary of visual object understanding this needs to be extended with art by at least two central qualities: First, it demands a viewer’s continuous (background) awareness of the “likeness” character of the artistic artefact (i.e. recognizing its true category status as a representation) [32], which has been described as a kind of metacognitive framing [87]. This awareness has been identified as a precondition for a twofold orientation on form (appearance) as well as content (meaning) as epistemologically separable domains [48, 81]. Secondly, to perceive something *as art* requires the presupposition its physical appearance is not arbitrary but deliberately follows from an (potentially highly obscure) artistic intention that guided its conception and production [78, 88]. Since this often cannot be directly deduced from the artefact itself (e.g. Warhol’s “Brillo Boxes” are visually indifferent from packing boxes), Bullot and Reber [36] proposed that cognizing art is linked to other artifactual object-categories, whose perceived identity is based on inferred properties (such as intended functionality or context of usage) as discussed in theories on psychological essentialism, see [89, 90]. Again, this outlines, when confronting art, the human mind sets corresponding mental processes somewhat apart from artlessness e.g. of faces or landscapes, which can be appreciated without cognitive mastering since they do not possess “aboutness” and or not relicts of human agency. Success in this mastering endeavour is often neither trivial nor guaranteed as cognition is genuinely challenged by polyvalent cues and a plurality of often equally valid interpretations [91] that, furthermore, can be reconsidered at any time.

The model contends that cognitive mastering activities strive towards mental closure and cognitive resolution in an attempt to restore mental coherence as the preferred state in our percepts and cognitions [67, 68, 92]. In line with earlier homeostatic drive theories [93–95] and a recent neuropsychological framework of visual processing [17] this reduction of tension is assumed to be phenomenally pleasing and to feed into the aesthetic pleasure during the aesthetic episode [78].

Cognitive mastering effects can be likened to what has been coined “aha!” or eureka effect [57, 96, 97], which designate the pleasurable moment of a sudden insight into a previously puzzling or incomprehensible artwork [43] (e.g. by reading a title that “explains it all”). However, in many situations an observer may derive at “aboutness” through a continually evolving process of cognitive elaboration, in which physical properties of an artwork are repeatedly re-evaluated in cycles of hypothesis generation and testing. As summarized by Danto [88]: “the artwork is a material object, some of whose properties belong to the meaning, and some of which do not. What the viewer must do is interpret the meaning-bearing properties in such a way as to grasp the intended meaning they embody” (p. 38). We propose that this distilling of meaning is typically achieved by iterative cycles of elaboration that may or may not be accompanied by intensive “aha!” moments. Why an elaboration-based mastering mechanism is implied in popular cognitive accounts of art perception e.g. [45, 49, 98] but has rarely been directly empirically investigated as a source of aesthetic pleasure can be easily explained with research’s primary focus on immediate preferences and neglect of time-domain–a critique already expressed in Fechner’s discussion of temporal gestalts (“Zeitgestalten”) of aesthetic pleasure that seems still valid [99].

### Implications for research in aesthetics

Our notions have three central implications: First, cognitive mastering constitutes a challenge-based response mechanism that is triggered by excessive processing demands prior to any later regulatory effects e.g. [100, 101]. Therefore, a certain degree of conflict, incongruity, or surprisingness is required to violate expectations and to provoke what Piaget [67] coined “cognitive disequilibrium”. This exemplifies why high fluency can be associated with quick, intuitive, and effortless processing [102] but fails to induce mastering-related reward effects, as, in a strict sense, only disfluency reflect novel information to the brain and require specific attentional orienting. Therefore, mastering activities may be especially relevant for the appreciation of disfluent entities. Moreover, mastering disfluent art may be especially pleasurable since coping of novel, conflicting, or ambivalent information reflects attainment of knowledge and learning, which is considered to be intrinsically rewarding and pleasurable itself [68, 92, 103–106].

Second, mastering of challenging art should essentially operate on a deliberate cognitively controlled level (of what has been coined “System 2” [107] or”reflective system“[108]) but cannot be expected at an implicit pre-reflective level as implied by several Gestaltists, psychophysical or computational approaches to aesthetic perception, see [109, 110]. This is substantiated by neuropsychological findings that novel events automatically capture attention [111] and that novelty detection–even at an early perceptual level–triggers subsequent attentional orienting [112]. Disfluency seems predestined to investing greater cognitive effort [102, 113, 114] since unfamiliar, ambiguous, or surprising (aesthetic) information should cause attention-binding mismatches that send information “upwards” the processing chain [115]. As noted by Alter, Oppenheimer and Epley [102] people respond to metacognitive difficulty by deeper cognitive processing and analytical reasoning. Moreover, a deliberate conscious state of awareness is constitutive for perception of art [116] in that sensuous experiences are not just “mere perceptual acts” but intentionally observed states that usually stay at the periphery of awareness [32]. As many aestheticians long presumed, not to interpret its physical properties is to not see the artwork at all [117]. Consequently, especially those properties that defy an immediate understanding should foster explicit awareness and encourage investing greater cognitive effort to reach an unapparent conclusion (if this fails, a disfluent artistic artefact will most likely appear as either poorly executed, incomprehensible, meaningless, or simply artless). This outlines why passive forms of enhanced mere exposure or visual familiarity that are known to affect implicit preference formation of a wide range of objects e.g. [118–121] may work for art under fairly limited constraints only [122] and may have a particularly limited scope for challenging art. Indeed, experimental evidence for mere exposure effects with art is noticeably tenuous and has revealed rather small effect-sizes or mixed results [122, 123]. This led Bornstein [124] to conclude in his metareview, mere exposure effects are inconclusive when paintings and drawings are the subject of study. Moreover, one may recall the mere exposure paradigm was initially proposed for evaluatively neutral stimuli [118], a condition that seems difficult to apply to challenging art, as disfluency experiences are genuinely negatively valenced [113].

Instead, as proposed by Martindale [125], when confronting art, perceived meaning seems to prevail as the most dominant factor–which may easily supress any mere exposure, familiarity, or perceptual fluency effects on a pre-reflective “retinal level”. This gets further apparent from the standpoint of a processing hierarchy model of aesthetic experiences [78] which predicts higher-level (“top-down” controlled) stages to overshadow lower-level (implicit perceptual) stages in their relative contribution towards affective pleasure. In line with these notions Biederman and Vessel [70] contended, repeated exposure for novel meaningful visual stimuli may be preference-enhancing only if this contributes to conceptual learning and therefore “the phenomenon of increased preference with exposure should be the exception rather than the rule” (p. 254).

Third, the rationale of a dynamical mastering mechanism parts way with so-called “optimal-arousal” models in the psychobiological tradition. Perhaps most prominently, Berlyne [39] proposed a converse U-shaped function between aesthetic appreciation and levels of arousal-inducing “collative” variables (e.g. novelty, conflict, and uncertainty) predicting that medium–but not excessive–levels to be most pleasurable. This arousal-based view that primarily focuses on object characteristics (and mostly disregards cognitively induced pleasure) has had a tremendous influence on scholars of aesthetics until today [77]. However, not only were Berlyne’s empirical predictions often difficult to sustain when applied to meaningful stimuli and art [126, 127] but also what defines an “optimum level” primarily depends on the situation and seems highly relative in its definition [68, 92]. Perceiving art may constitute a very special situation, that deviates in important aspects from that of every-day situations: First, Kant introduced the notion that aesthetic perception is characterized by a state of disinterestedness (“uninteressiertes Wohlgefallen”) [128], thus deprived to fulfil immediate utilitaristic goals or survival needs. From a neuropsychological perspective this aesthetic attitude has been described as “liking” without “wanting” [129]. Therefore, perceiving art may adjust expectations towards increased thresholds of mental stimulation, particularly since cognitive effort and high-levels of arousal are often deliberately sought for [130]. Second, art symbolizes content in a hypothetical manner [36, 131], which enables a viewer to mentally decouple experiences from actual real-world events [1, 72]. In this sense, experiences of (pictorial) art have been described as an act of “pretense activity” [61] that liberates cognition from outcomes of the natural environment and allows perceiving reality from a safe distance [1, 62, 132]. Hence, contrary to Berlyne’s prediction, far from optimal-arousal levels may be enjoyable and aesthetically pleasing as “no real threat emanates from a canvas”. Due to these conceptual and empirical limitations particularly for art-experiences (for further points of critique see Silvia [77]), this study’s theoretical grounding was oriented on alternative accounts.

### The present study

The present study aimed to reveal mental processing conditions that may explain how mentally challenging art that is initially rejected becomes preferred. This question was theoretically framed by assumptions of our model on cognitive mastering according to which elaboration of conveyed meaning is particularly crucial for disfluent art to be experientially rewarding. Depictions of portraits paintings of renowned artists served as stimulus material that empirically revealed two opposing potentials: One high in processing fluency (subsequently referred to as *fluency* portraits) and one high in cognitive stimulation (subsequently referred to as *mastery* portraits). Thus, rather than differing in a single aspect, we provided two contrasting types of portraits that differed in their overall mental accessibility (challengingness). The portraits underlying dimensional structure was empirically obtained from an extensive rating study via principal component analysis (PCA). This made apparent the spectrum of perceived qualities of the two sets that were initially compiled based on ratings of (low and high) *atypicality*.

To utilize portraits combined a few methodological advantages: First, all stimuli were identical in subject matter, which enabled to associate variations of depiction between portraits to differences in mental challengingness. Second, a bulk of research demonstrated that faces appear to be the single most important domain of human object-recognition with brain-mechanisms underlying face-perception being particularly refined e.g. [133, 134]. As pointed out by Tanaka [135], in face-perception everybody is an expert. This “wiredness” towards faces should enable to recognize subtle artistic nuances in the depiction of a sitter`s face or in conveying expressive-emotional information [132, 136, 137] even without formal training in the arts. Third, portrait painting is a major (and possibly in several periods dominant) genre in the history of painting with depictions of human faces being traceable to 27, 000 year-old cave paintings in the *Vilhonneur grotto* in France [138]. This significance within the fine arts should contribute to the ecological validity of potential findings [139].

In order to capture dynamical preference changes we applied a suitable test-paradigm that was previously applied in a number of studies on aesthetic appreciation of designed artefacts [140–142]. In this within-subjects paradigm stimuli are repeatedly evaluated on numerous rating scales, which foster deeper elaboration of stimulus targets, and preferences measured before and afterwards this phase. This allowed revealing how preferences were affected by active elaboration conjointly for *mastery* and *fluency* portraits over time and to compare results with passive perceptual processing conditions.

In particular, in Experiment 1, preference changes for *fluency* and *mastery* portraits in a repeated evaluation condition were compared with a familiarization condition matched in viewing time. In Experiment 2, we crosschecked results obtained in the repeated evaluation task with an additional control condition, in which viewing times were significantly shortened and mere exposure effects predicted to be more likely [19, 124]. In Experiment 3, a repeated evaluation follow-up task was carried out with a novel and more extensive pool of portraits. This enabled to corroborate the robustness of findings of Experiment 1 and to validate the portraits suitability for a subsequent task. Experiment 4 consisted of a generalization task, in which different exemplars of the same artists were shown between evaluation and test phase. Thereby, we could exploratively investigate whether elaboration effects would depend on the very exemplar (as predicted by a cognitive mastering hypothesis) or alternatively, if mastering effects would generalize within the same artist-specific style (as predicted by a perceptual learning hypothesis).

The following predictions were tested: First, we expected *mastery* portraits to be initially preferred at low levels due to aversively strong stimulus-uncertainty or lack of (self-assessed) comprehension. Second, positive preference shifts should depend on the interim processing-condition and selectively show-up for *mastery* portraits. If preferences are mediated by an elaboration-based mastering mechanism, then preference increases for *mastery* portraits were expected in the repeated evaluation conditions only. Alternatively, if initial rejection of *mastery* portraits results from a lack of perceptual familiarity or a perceptual mismatch between stimulus appearance and its underlying memorial representation, then increasing visual exposition (in the familiarity and mere exposure conditions) should equally increase their preferences. Moreover, preference effects could depend on perceptual learning as predicted by a visual adaptation hypothesis [143, 144]. This posits a dynamical accommodation of memorial prototypes with repeated exposure, a process Webster and MacLeod [145] coined “renormalization” (p. 1707). In Experiment 4 this hypothesis was directly tested. If preference effects rely on such visual adaptation towards artist-specific styles, some flexibility of this mechanism was expected that should allow for preferences to generalize onto novel exemplars of the same prototypical appearances. Otherwise, if preferences are mediated by conceptual processing of semantic expressive content–unique to each exemplar–preference effects should depend on the very “token” and not generalize across “types”. Moreover, a dependence of effects on the level of unique identity would be further indicative of a “top-down” controlled meaning-driven process, whose characteristics have been described by Kandel [132] as idiosyncratic.

A study on cognitive processes with art should consider a viewer´s level of art-expertise as a potential key-moderator [78, 98, 127]. In particular, it has been shown that resolution of ambiguity, extraction of information, or problem solving skills are conjointly traceable towards the possession of art-specific knowledge structures [146–150]. In line with these findings the model predicts greater art-expertise to expand mastering competences [78]. To statistically control preferences to be shaped and”biased” by such interindividual differences we considered each participant’s amount and sophistication of art-specific knowledge. In particular, a questionnaire was applied that assessed explicit remembering of canonical paintings (by asking for corresponding artists’ names, titles, and art-related terms) as well as tacit knowledge (such as familiarity with paintings that allows for successful recognition without explicit retrieval of corresponding terms). Previously, this questionnaire was provided in several studies on art-perception e.g. [151–153] and enabled to obtain an overall index of the aesthetic literacy.

## General Method

### Ethics Statement

Experiments of this study were administered in full compliance with ethical standards of the Faculty of Psychology, University of Vienna and the Department of Education and Psychology of the Free University of Berlin. According to the Austrian Universities Act (UG2002) only medical universities were required to appoint ethics committees for clinical tests, application of medical methods, and applied medical research. Likewise were the legal requirements at the Free University of Berlin. Therefore, no ethical approval was required for the present study. All participants provided written informed consent and could withdraw their participation at any point without further consequences. The only demographic information of age and sex was anonymously assessed. Participants were recruited by written notice in several university departments and offered partial course credit (for undergraduate programs in Psychology), or, alternatively, a compensation fee of six Euros.

### Stimuli: Selection

All stimuli were depictions of real portrait paintings by renowned artists. The portraits were initially selected based on (high vs. low) *atypicality* ratings in two pilot-studies but revealed a more complex dimensional structure in a subsequently carried out rating study (see below). A first pool of portraits was applied in the initial tasks (Experiment 1 and 2). A second more encompassing pool was applied in the follow-up tasks (Experiment 3 and 4).

#### Stimulus pool 1

Ten portraits were selected based on results of a pre-study. As selection criterion served the scale “how unusual and extraordinary is this portrait?” that operationalized the variable “originality by virtue of introducing new ideas” [154], p.587. Originally labelled “innovativeness” this variable proofed suitable to identify designed artefacts that are prone to dynamical preference changes after repeated exposure [140–142, 155, 156]. Considering its close negative correlation to novelty and typicality in these previous studies, we will denote this variable with the less connotative term *atypicality*. Participants in the pre-study were 31 students (25 females, mean age: 22.4 years) from the University of Vienna. In a group session, participants were asked to rate a pool of 34 portraits according to their degree of *atypicality* on a 7-point Likert scale that were successively presented via video-projector. The order of portraits was pseudo-randomized. To ensure their central motif (depictions of a sitter’s face) was comparable in size and figure-ground ratio, some image areas were cropped. For each of the two sets, the five portraits with highest and lowest means for *atypicality* were chosen. Set A consisted of low *atypicality* portraits (*M* = 3.00; *SD* = 1.33) by DaVinci, Fragonard, Gertsch, Janssen, and Manet. Set B consisted of high *atypicality* portraits (*M* = 6.86; *SD* = 1.58) by Baselitz, Jawlenski, Klee, Monet and Picasso. Differences in *atypicality* between sets were significant, *t*(30) = 10.10, p < .01, η_p_^2^ = .77. See S1 Fig for an overview.

#### Stimulus pool 2

A second stimulus pool consisted of 20 portraits. None of them was included in the first pool. Based on results of a pilot study we selected from each artist pairs of two that closely resembled each other stylistically. Again, some image sections were cropped. Ten students (six female) from the University of Vienna, mean age of 23.3 years (range: 20–30 years) took part in a rating study analogously to the above-described one. Pairs of portraits with highest and lowest mean ratings of *atypicality* were selected. Two sets were compiled, each containing ten portraits by five different artists. The first set of portraits with lowest *atypicality* ratings (*M* = 2.6, *SD* = 1.4) comprised portraits by Manet, Raffael, Rossetti, Watteau, and Schad. The second set with highest means of *atypicality* (*M* = 5.7, *SD* = 1.0) consisted of portraits by Baselitz, Brown, Jawlenski, Kluge, and Lüpertz. See S2 and S3 Figs for an overview.

### Stimuli: Principle component analysis

All 30 portraits were further submitted to an extensive rating study to reveal the factor structure underlying perception of portraits by means of a PCA. This seemed crucial since differences in *atypicality* appeared to be confounded with multiple stimulus dimensions on a perceptual as well as conceptual level. For the item generation an extensive body of literature from the field of empirical aesthetics was considered, with a particular focus on Berlyne’s [94] seminal studies and Hager et al.’s [153] recent survey on art-reception. The final 36 items referred to descriptive, affective, semantic, expressive, and style-related qualities, as well as to normative cultural aspects.

#### Subjects

Twenty-seven psychology students from the University of Vienna were recruited. The mean age of participants was *M* = 26.2 years (range: 24–31 years).

#### Procedure

The questionnaire was provided in a group session as a paper and pencil test. Each participant had to rate all 30 portraits according to 36 items, resulting in 1080 responses per person. Portraits were presented consecutively via overhead projector for 4 minutes each.

#### Results and Discussion principle component analysis

Here we give a short summary of the PCA’s main results (for a more detailed discussion see S1 Text). Findings revealed a coherent and interpretable factorial structure, due to relatively low cross-factor-loadings of all marker items. Most notably, two factors–*accessibility* and *cognitive stimulation*–dominated perception of portraits, followed by *zeitgeist*, *stylistic components*, *affective valence*, *coping potential*, and *complexity*. The eigenvalues of the first two factors put together alone were 15.2, explaining 46% of variance of the data, which emphasized their overall importance. Table 1 gives an overview of the final set of variables and corresponding factors. The first factor *accessibility* subsumed fluency-related qualities that influenced the “processability” of portraits on a perceptual and conceptual level. In particular, this factor had the marker item *comprehensibility* (factor loading = -.70) and was related to the variables *unambiguity*, *attractiveness*, *order*, *realism*, *abstraction* (negatively correlated), *determinacy*, *emotional clarity*, and *typicality* amongst others. Its dominance made apparent that fluency-experiences derived from the portraits were strongly reflected in their perception. The second factor *cognitive stimulation* subsumed variables that mentally excite and foster cognitive engagement with the portraits. It had the marker item *idiosyncrasy* (factor loading = .82), and was related to the variables *expressiveness*, *intentionality*, *imagination*, *interest*, *innovativeness*, *importance of style* and *atypicality*. This factor mirrored findings of a recent art reception survey [153].

**Table 1:** Results of the Principal Component Analysis. Items and item parameters separated by PCA factors (with factor-loadings > .5) and post-hoc comparisons between *fluency* and *mastery* portraits.

PCA factor	Item	Loading	*M* (*SD*) for Fluency portraits	*M* (*SD*) for Mastery portraits	*Contrast*
1. Accessibility	*Comprehensibility (comprehensible—incomprehensible)*	-.702	2.73 (0.15)	4.35 (0.16)	**
	Unambiguity (unambiguous—ambiguous)	-.697			
	Attractiveness (The sitter´s face is attractive)	-.692			
	Order (orderly—not orderly)	-.678			
	Realism (Compared to a real face this is a realistic mode of depiction)	.678			
	Abstraction (abstract—concrete)	.649			
	Determinacy (It is easy to decide on the sex of the sitter)	.644			
	Emotional clarity (The facial expression of an emotion is clearly determinable)	.582			
	Typicality (This portrait is typical compared to a classical portrait)	.579			
	Roundness (round—angular)	-.569			
	Liking (I like this portrait)	.569			
2. Cognitive stimulation	*Idiosyncrasy (This portrait reflects an individual way of the artist to perceive single objects or the world as a whole)*	826	4.61 (0.26)	7.06 (0.17)	**
	Expressiveness (This portrait is used by the artist to express his feelings or emotions)	.817			
	Intentionality (This portrait reflects specific beliefs and thoughts of the artist)	.795			
	Imagination (This portrait contains imaginations and phantasies of the artist)	.790			
	Interest (interesting—uninteresting)	-.636			
	Innovativeness (innovative—non-innovative)	-.519			
	Importance of style (stylistically salient—stylistically ordinary)	-.517			
	Atypicality (This portrait is unusual and extraordinary)	.514			
3. Zeitgeist	*Aesthetic Norms (This portrait reflects aesthetic norms and conventions that characterize society and period at that time)*	.832	6.26 (0.17)	3.93 (0.24)	**
	Conventions (This portrait contains beliefs and stylistic conventions that characterize society and period at that time)	.810			
4. Stylistic components	*Shapes (In this portrait shapes are of particular importance)*	.779	5.32 (0.23)	6.23 (0.15)	**
	Lines (In this portrait lines are of particular importance)	.748			
	Colours (In this portrait colours are of particular importance)	.635			
5. Affective valence	*Tone (warm—cold)*	-.720	3.34 (0.14)	4.42 (0.09)	**
	Hue (sombre—bright)	.703			
	Mood (tense—calm)	.430			
6. Coping	*Coping (I am able to deduce a deeper meaning from this portrait)*	.819	2.90 (.30)	3.24 (.28)	*ns*
	Meaningfulness (This portrait contains a deeper meaning)	.646			
	Familiarity (I am familiar with this portrait or the artist)	.600			
7. Complexity	*Richness of detail (contains many details—contains few details)*	-.783	4.10 (.16)	4.33 (.17)	*ns*
	Simplicity (simple—complex)	.691			

*Note*: Marker items are printed in italics

** significant differences at *p* < .0001.

Critically, both portrait sets showed a strict complementary factorial pattern for the two main factors, as revealed by further Analyses of Variances (ANOVAs). Accordingly, *fluency* portraits were marked by significantly higher degrees of *accessibility* and significantly lower degrees of *cognitive stimulation*, while *mastery* portraits showed the inverse pattern (see Table 1). This corroborated that *fluency* and *mastery* portraits formed two coherent clusters that were distinguished by a complementary impression of how mentally challenging they appear.

In sum, although *atypicality* served as their initial selection criterion, differences between the portrait sets could not be confined towards this variable. Instead, factor loadings revealed that *atypicality* had a high discriminatory power to distinguish between the more fundamental *mastery* and *fluency* related (meta-) dimensions that each encompassed a spectrum of associated variables. No significant differences between portrait pools one and two on any of the seven factors were found.

### Experiment 1: Repeated evaluation versus familiarity condition

We applied a repeated evaluation task, in which participants rated portraits one-by-one on numerous evaluation scales and compared effects on liking between *fluency* and *mastery* portraits. A familiarity task served as the control condition, in which portraits were presented without any evaluation instruction but with the same exposure rate (of 20 presentations per portrait) and a matched average presentation time (of 2,900 ms) to ensure processing times in both conditions were comparable.

#### Subjects

Forty-eight participants (36 females) from the Free University of Berlin enrolled in different study programs participated in the experiment. Participants mean age was 25.5 years (range: 20–28 years). All participants received a compensation of six Euros for participation and were tested individually.

#### Stimuli

Ten portraits with five *fluency* and five *mastery* portraits of the first stimulus pool (see S1 Fig).

#### Apparatus

The experiment was administered using PsyScope PPC 1.2.5 (Cohen, MacWhinney, Flatt, & Provost, 1993) on Apple eMac and iMac computers. Stimuli were centrally presented on a 17-inch monitor at a size of 652 x 532 pixels, 72 dpi and a screen resolution of 1024 x 768 pixels.

#### Procedure

Experiment 1 consisted of two (test-retest) evaluation phases and an interim phase, in which elaboration of portraits was either encouraged or not depending on the experimental condition. In an initial exposition phase that should sensitize participants to the inherent variations, all ten portraits were simultaneously presented for 10,000 ms. In the first evaluation phase (t1), participants were asked to spontaneously rate each of the ten portraits on four scales in the following order: *liking*, *atypicality*, *complexity*, and *realism*. Only liking and atypicality ratings were further analysed (as in [140]). In the second part, labelled t2, half of the subjects were randomly assigned to a repeated evaluation condition and the other half to a familiarity condition. Participants in the repeated evaluation condition were asked to rate each portrait according to 20 different scales in a block-wise way, resulting in 200 experimental trials. More specifically, each portrait was rated according to ten adjective scales and ten statements (see S2 Text for a complete list). In each of the 20 rating blocks portraits were shown in random order. Participants in the familiarity condition were instructed to “attentively look at the portraits”, each of which appeared for 2,900 ms at a time. Otherwise the trial-scheme was identical to the repeated evaluation condition (each portrait was presented 20 times in a block-wise and randomized manner resulting in 200 trials). The time interval equalled the average (self-controlled) viewing-time of participants in the repeated evaluation condition. Subsequently to the interim phase participants were asked to complete a second evaluation phase t2 that comprised of the same four ratings as in t1. All ratings were assessed using 7-point Likert scales, ranging from 1 (*not at all*) to 7 (*very much*). No time constraints were set for giving these ratings. After the experimental sessions, each participant conducted a paper-and-pencil test to assess the level of art-expertise. A complete experimental session lasted about 45 minutes.

### Results and Discussion Experiment 1

#### Ratings of Liking

Of main interest were the dynamic changes in liking for mastery and fluency portraits before and after having been presented in the repeated evaluation or familiarity condition. As a quasi-experimental factor, we considered the influence of art-expertise, operationalized by a median-split based on each participant´s art-expertise sum score. The resulting cell-assignment to each condition was even (with 12 relative low vs. 12 relative high expertise participants). Mean ratings of liking sampled over participants were analysed and are shown in Table 2. A four-way mixed-design repeated measurement ANOVA with phase (t1, t2), set (mastery, fluency) as within factors and condition (repeated evaluation, familiarity) and expertise (low, high) as between factors was carried out with main effects for phase, F(2,44) = 7.84, p = .0076, η_p_^2^ = .151, set, F(2,44) = 28.44, p < .0001, η_p_^2^ = .393, and expertise, F(2,44) = 6.57, p = .0139, η_p_^2^ = .130, as well as significant interactions between, set, and expertise, F(2,44) = 4.55, p = .0385, η_p_^2^ = .094, and phase and set, F(2,44) = 5.75, p = .0207, η_p_^2^ = .116 as well as a marginally significant interaction between phase and condition, F(2,44) = 3.91, p = .0544, η_p_^2^ = .082, n.s. Most important, these effects were qualified by a four-way interaction between all factors, F(2,44) = 4.12, p = .0484, η_p_^2^ = .086. No other effects were significant. The main effect of expertise indicated its systematic influence, why we will report results separately for each expertise group.

**Table 2:** Liking ratings for Experiment 1–4. Mean Liking ratings for *fluency* and *mastery* portraits, depending on experimental condition, separated for groups of low and high art-expertise and by phase. Standard deviations are noted in brackets.

	Repeated Evaluation		Familiarization		Mere Exposure		Replication		Transfer	
	*Low art-expertise groups*									
	**t1**	**t2**	**t1**	**t2**	**t1**	**t2**	**t1**	**t2**	**t1**	**t2**
Fluency	4.30 (0.68)	4.42 (0.98)	4.02 (0.87)	4.22 (0.87)	4.04 (0.93)	4.30 (1.01)	4.43 (1.18)	4.13 (1.01)	4.62 (0.82)	3.60 (1.12)
Mastery	2.72 (0.86)	3.78 (1.09)	3.00 (0.76)	2.90 (0.65)	3.06 (0.80)	3.27 (0.88)	2.48 (0.96)	3.25 (1.34)	3.05 (0.98)	3.28 (1.35)
	*High art-expertise groups*									
	**t1**	**t2**	**t1**	**t2**	**t1**	**t2**	**t1**	**t2**	**t1**	**t2**
Fluency	4.27 (1.01)	4.18 (0.74)	4.50 (0.81)	4.25 (1.05)	4.37 (1.12)	4.55 (1.12)	3.78 (1.23)	4.07 (1.04)	5.12 (0.98)	4.25 (0.83)
Mastery	3.57 (.54)	3.92 (0.62)	3.68 (0.86)	4.08 (0.96)	3.17 (1.39)	3.42 (1.20)	4.00 (0.86)	3.92 (0.89)	3.17 (1.36)	3.02 (1.20)

*Note*: Post-hoc assignment to groups of relative low and high art-expertise groups was even (*N* = 12) except for the mere exposure condition (low expertise: *N* = 11, high expertise: *N* = 13)

#### Ratings of Liking: Art inexperienced participants

First, we analysed results for the subsample of 24 art-inexperienced participants, based on 12 participants in the repeated evaluation condition and familiarity condition each. For a sample of 12 participants the sensitivity calculated with G*Power3 (for a mixed ANOVA, two measures, and α = 0.05) revealed that an effect size of *f* = .573 (medium-sized as defined by Cohen, 1988) could be detected. We considered the sample size as appropriate for the expected effects since previous studies on *atypicality* (see *innovativeness*) that utilized a similar repeated evaluation design were reported to be of medium size as well [142, 157]. Table 2 shows that initial appreciation (t1) for *mastery* was lower than for *fluency* portraits. After a repeated evaluation phase preferences for *mastery* portraits increased, while appreciation for *fluency* portraits remained stable over time. No such preference gains for *mastery* portraits were observed after the familiarity task. In order to analyse these effects, a mixed-design repeated measurement ANOVA with *phase* (t1, t2) and *set* (mastery, fluency) as within-subjects factors and *elaboration* (repeated evaluation, familiarity) as between subjects factor was performed on ratings of liking as the dependent variable. The ANOVA revealed significant main effects for *phase*, *F*(1,22) = 11.77, *p* = .002, η_p_^2^ = .348 and *set*, *F*(1,22) = 44.20, *p* < .0001, η_p_^2^ = .668 as well as a significant interaction between *phase* and *condition*, *F*(1,22) = 8.39, *p* = .0008, η_p_^2^ = .276. Importantly, these effects were qualified by a three-way interaction between *phase*, *set* and *condition*, *F*(1,22) = 5.66, *p* = .027, η_p_^2^ = .205. We further analysed this three-way interaction by testing Bonferroni adjusted simple main effects of *phase* for both conditions of *elaboration* and both conditions of *set*. Critically, this analysis revealed that the only significant effect of *phase* was found in the repeated evaluation condition for *mastery* portraits, *p* < .0001. This means, that preference increases between t1 and t2 were restricted to the repeated evaluation condition and selectively showed up for *mastery* portraits. There were neither effects of *phase* for *fluency* portraits in the repeated evaluation condition, *p* = .6439, nor effects of *phase* in the familiarity condition for either *mastery* portraits, *p* = .6307, or *fluency* portraits, *p* = .4303. Bonferroni adjusted post-hoc tests indicated that *mastery* portraits were significantly less preferred compared to *fluency* portraits in both conditions at t1 and at t2 in the familiarity condition (*p*s < .001). This difference diminished at t2 in the repeated evaluation condition where it showed-up only tendentially (*p* < .060, *n*.*s*.). In other words, after elaboration but not mere visual exposition of portraits the initially high discrepancy in liking between both sets nearly converged.

#### Ratings of Liking: Art experienced participants

Results for the relative art-experienced group were likewise tested with Bonferroni-adjusted simple main effects. This group did not show a selective increase for mastery portraits in the repeated evaluation condition, p = .128, but (unexpectedly) in the familiarity condition, p = .044. Furthermore, simple main effects showed that art-experienced participants significantly preferred fluency to mastery portraits in both conditions at t1 (i.e. familiarity condition, p = .019, repeated evaluation condition, p = .042). However, compared with the art-inexperienced group preferences for mastery portraits were significantly higher in both conditions at t1 (i.e. familiarity condition, p = .035, repeated evaluation condition, p = .010), as well as at t2 in the familiarity condition, p = .002. The lack of simple main effect between both groups for mastery portraits in the repeated evaluation condition at t2 was due to the preference increase of art-inexperienced participants. This demonstrated that expertise-dependent preference-differences for mastery portraits vanished after elaboration.

#### Ratings of Atypicality

The aim of analysing ratings of *atypicality* was twofold. First, this served as a treatment check, to reveal if both portrait sets differed in their degree of perceived *atypicality*. Second, this allowed to determine whether ratings of *atypicality* remained stable over time and thereby rule-out a potential confound between liking and *atypicality*. A mixed design repeated measurement ANOVA with *phase* (t1, t2) and *set* (mastery, fluency) as within factors and *condition* (repeated evaluation, familiarity) as between factor on *atypicality* ratings as the dependent variable revealed a main effect of *set*, *F*(1,22) = 97.12, *p* < .0001, η_p_^2^ = .815. No other effects or interactions were significant. This confirmed that both sets clearly differed in perceived *atypicality*. The lack of main effects and interactions with *phase* indicated that perceived *atypicality* was stable regardless of the elaboration condition. Analyses of variance for the whole sample and *expertise* as an additional quasi-factor revealed no main effects or interactions of *expertise* on *atypicality* ratings. Thus, art-expertise did not significantly alter perceived levels of *atypically* of the two portraits sets.

#### Summary

In sum, results of Experiment 1 (for inexperienced viewers) showed a preference increase for *mastery* portraits that selectively emerged in a repeated evaluation, but not a mere familiarization condition, and could, therefore, not be traced towards a repeated measurement procedure or towards enhanced perceptual familiarity per se. The unexpected finding of increased liking for *mastery* portraits observed for participants with relative high art-expertise in the familiarity condition provided a first hint that the possession of art-relevant knowledge-structures might have enabled for a self-induced elaboration-based mastering that does not depend on external encouragement (see General Discussion).

### Experiment 2: Mere exposure task as additional control condition

A classical mere exposure task was applied in Experiment 2 that served as a second control condition for the repeated evaluation condition of Experiment 1. Considering that mere exposure effects are most strongly pronounced during brief presentation times [124] an exposure duration of 500 ms was chosen. Since mere exposure “needs no inferences” and constitutes a passive (non-reflective) preference mechanism [158–160] this task was suitable to further corroborate whether or not the observed repeated evaluation effect relied on *active* intentional mental processing. Specifically, the following hypotheses were tested: If the preference mechanism requires mental regulation on a cognitive level, mere exposure effects were not expected and should be clearly dissociable from effects of the repeated evaluation task (Experiment 1). Moreover, previous research on time demands during aesthetic episodes demonstrated that exposure times fewer than 1000 ms restrict the extraction of information from a painting and block deeper elaboration (e.g. of metaphorical content, self-related meaning) [161]. Therefore, due to stricter time constraints, self-initiated elaboration effects of art-experienced viewers (as suggested by results of the familiarity condition of Experiment 1) were not expected. Alternatively, if the preference mechanism is primarily regulated on an implicit pre-reflective level results should demonstrate a mere exposure effect for *mastery* portraits. Again, the relative level of participants’ art expertise was considered for potential moderations.

#### Method

Stimuli, apparatus, and procedure were identical to the familiarity task in Experiment 1 except for a shortened stimulus presentation time of 500 ms.

#### Subjects

24 participants (21 female) from the University of Vienna participated for partial course credit. Mean age was 21.9 years (range: 19–28 years).

### Results and Discussion Experiment 2

#### Ratings of Liking

**Fig 1:**
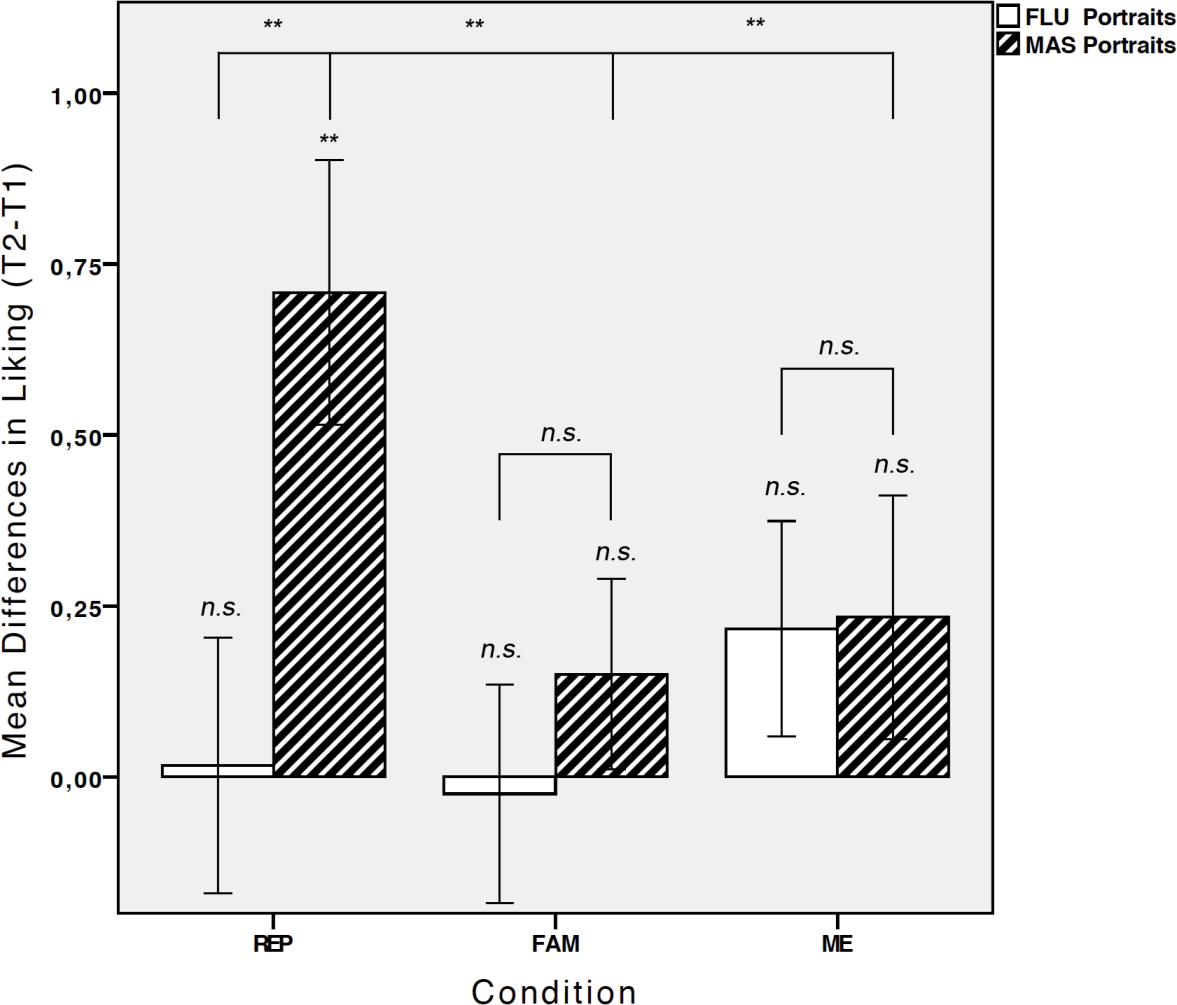
Liking for *fluency* and *mastery* portraits for Experiment 1–3. Mean differences in ratings of liking (t2-t1) for *fluency* (FLU) and *mastery* (MAS) portraits separately for the repeated evaluation (REP), familiarity (FAM), and mere exposure (ME) tasks. Error bars indicate +/- 1 SE. Asterisks depict differences between phases (t1, t2) or portraits sets (*fluency*, *mastery*) using SMEs (*p < 0.5, **p < 0.01).

First, we report the results for the mere exposure task. A 2x2x2 mixed-design repeated measurement ANOVA with phase (t1, t2), *set* (mastery, fluency) as within factors and *expertise* (low, high) as between-subjects factors was carried out on ratings of liking sampled over participants as the depended variable. The only significant effect was a main effect of *set*, *F*(1,22) = 17.81, *p* < .0001, η_p_^2^ = .447 indicating that *fluency* were preferred over *mastery* portraits at both phases (t1 and t2). Thus, results provided no evidences for a mere exposure effect. To crosscheck results with the repeated evaluation condition of Experiment 1, we analysed results of both tasks conjointly. Therefore, a four-way mixed-design repeated measurement ANOVA was carried out with *phase* (t1, t2), *set* (mastery, fluency) as within factors and *expertise* (low, high) as well as *condition* (repeated evaluation, mere exposure) as between factors. This revealed main effects for *phase*, *F*(2,44) = 8.79, *p* = .0049, η_p_^2^ = .665, and *set*, *F*(2,44) = 34.33, *p* < .0001, η_p_^2^ = .438 and an interaction between *phase* and *set*, *F*(2,44) = 4.92, *p* < .0318, η_p_^2^ = .101. Critically, these effects were qualified by a three-way interaction between *phase*, *set*, and *condition*, *F*(2,44) = 4.57, *p* = .0381, η_p_^2^ = .094. No other effects were significant. Fig 1 depicts the three-way interaction that mirrored the pattern of results of Experiment 1.

This demonstrated that preference enhancements for *mastery* portraits were restricted to the repeated evaluation condition and could not be found in the mere exposure condition.

#### Ratings of Atypicality

A 2x2 repeated-measures ANOVA for ratings of *atypicality*, with *phase* (t1, t2) and *set* (fluency, mastery) as within subjects factors revealed a main effect of *set*, *F*(1,23) = 87.06, *p* < .0001, η_p_^2^ = .791, and an interaction between both factors, *F*(1,23) = 4.59, *p* = .0430, η_p_^2^ = .166. While *mastery* portraits were generally perceived as highly atypical compared to *fluency* portraits, the interaction was due to a slight decrease in ratings of *atypicality* for the former and marginal increase for the latter towards the second measure. Importantly, at both phases differences in *atypicality* between sets were clearly perceived, at t1, M_*diff*_ = 2.92, *t*(23) = -10.01, *p* < .0001 and at t2, M_*diff*_ = -2.34, *t*(23) = -7.06, *p* < .0001.

#### Summary

Experiment 1 and 2 suggested, when elaboration is experimentally fostered in the repeated evaluation condition or potentially self-initiated by art-experienced viewers in the familiarity condition, preferences for *mastery* portraits increased. On the contrary, when participants were merely visually exposed to the portraits (in the familiarity and mere exposure conditions) or the time frame is expectable to restrict self-initiated elaboration, repeated exposure of portraits did not increase their liking. Findings thereby strongly suggested that underlying preference mechanism could not be traced towards enhanced perceptual familiarity, or mere exposure, as passive (implicit) forms of mental processing regulation. Instead, findings were in line with the idea of a cognitively driven preference mechanism for challenging portraits as predicted by our elaboration-based mastering hypothesis.

### Experiment 3: Repeated evaluation replication task

To reveal whether findings of Experiment 1 were replicable with a novel and more encompassing pool of portraits, in Experiment 3 we probed the critical repeated evaluation condition again. The objective for this task was twofold: First, we aimed to corroborate whether the observed effect is stable and robust, which seemed crucial since the number of portraits utilized in the initial task was relatively small. Moreover, obtaining the same findings with a different stimulus pool would rule out a potential response bias that rating-scales had one-sidedly favoured impression formation for specific portraits in Experiment 1. Second, in case the presumed effect could be reconfirmed this would qualify the pool of portraits to be applied in a subsequent experimental task, which enabled to test the corresponding preference mechanism in an alternative manner.

#### Subjects

24 participants (18 female) from the University of Vienna participated for partial course credit. Mean age was 21.0 years (range: 19–26 years). None of the participants took part in the previous experiments of this study.

#### Stimuli and apparatus

The second portrait pool was used consisting of 20 portraits from 10 different artists (see S2 and S3 Figs). The 10 *mastery* portraits were subdivided into set A and B, with each containing one of the two portraits from the 10 artists–while the *fluency* portrait set was analogously subdivided into set C and D. None of the portraits were applied in Experiment 1 or 2. The apparatus remained the same as described in Experiment 1.

#### Procedure

This repeated evaluation task was nearly identical to Experiment 1. Again, an initial exposition phase, in which all portraits were conjointly presented for 10,000 ms, preceded the task. In a test-retest design manner three consecutive phases followed: In the first phase (t1) participants had to rate five *fluency* and five *mastery* portraits according to the scales *liking*, *atypicality*, *realism* and *complexity* (with only the former two being analysed). Participants were either presented subsets A and C or subsets B and D (as each participant saw only 10 out of 20 portraits). The assignment of participants to this subset-condition was randomized and balanced. The following repeated evaluation phase comprised of the same rating block as the one of Experiment 1 (with 20 rating scales on which each of the ten portraits was evaluated, one-by-one, resulting in 200 experimental trials). In the last phase (t2) participants had to rate all portraits again according to *liking* and *atypicality* (as well as *realism*, and *complexity*). As before, all ratings were given via 7-point Likert scales.

### Results and Discussion Experiment 3

#### Ratings of Liking

First, we ran a 2x2x2 mixed ANOVA, with *phase* (t1, t2) and *set* (mastery, fluency) as within-subjects factors and *expertise* (low, high) as between-subjects factors. The main effect of *set* was significant, *F*(1,22) = 6.28, *p* = .0201, η_p_^2^ = .222 and was qualified by an interaction between *set* and *expertise*, *F*(1,22) = 6.90, *p* = .0154, η_p_^2^ = .239. Moreover, a significant three-way interaction between the factors confirmed that *expertise* mediated the central interaction between *set* and *phase*, *F*(1,22) = 7.48, *p* = .012, η_p_^2^ = .254. This made separated analyses for the two expertise groups reasonable, which allowed for a streamlined presentation of results.

#### Ratings of Liking: Art inexperienced participants

**Fig 2:**
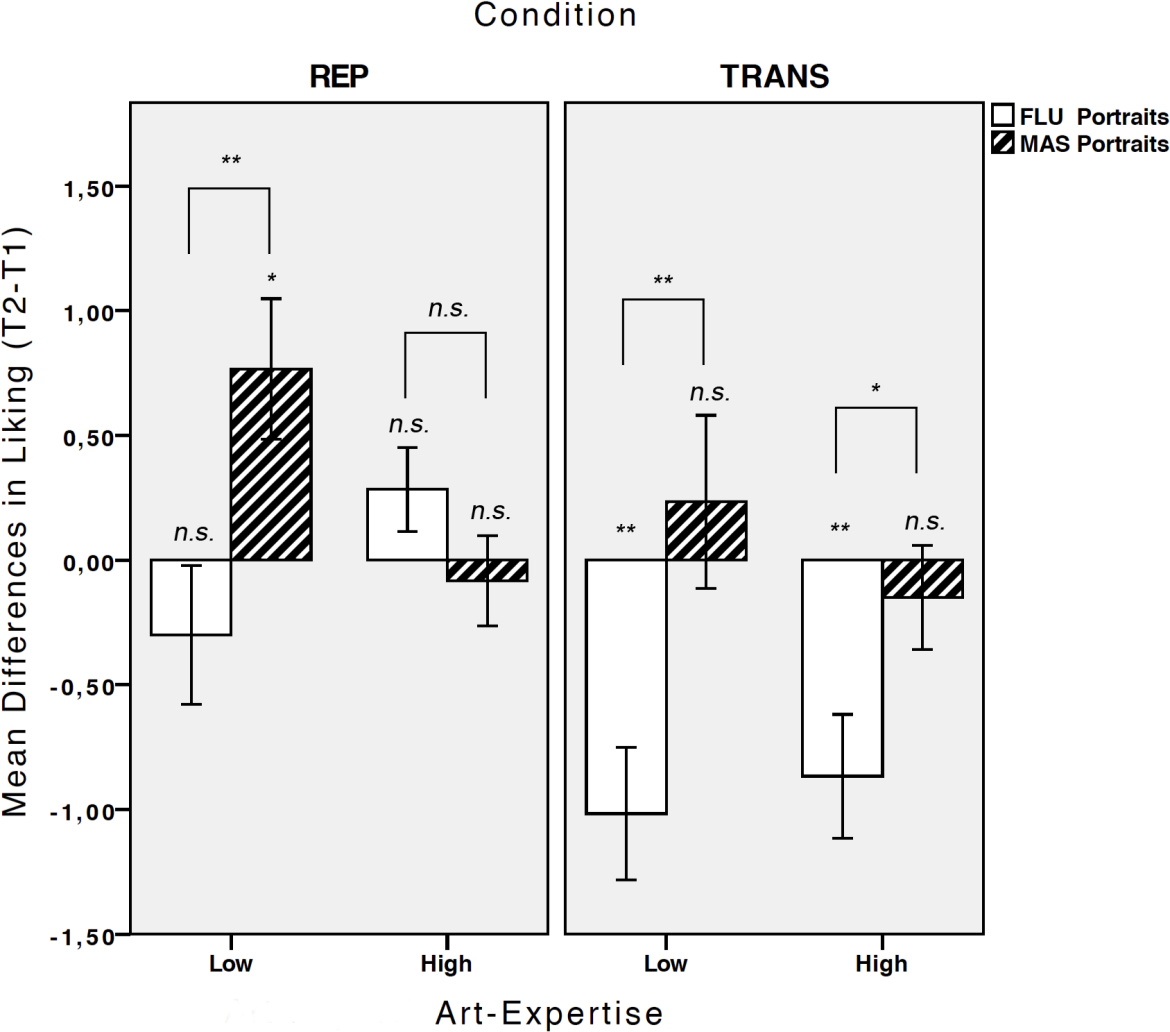
Liking for *fluency* and *mastery* portraits for Experiment 4&5. Mean differences in ratings of liking (t2-t1) for *fluency* (FLU) and *mastery* (MAS) portraits separately for repeated evaluation replication (REP) and repeated evaluation transfer (TRANS) tasks, separated by groups of low and high art-expertise. Error bars indicate +/- 1 SE. Asterisks depict differences between phases (t1, t2) or portraits sets (*fluency*, *mastery*) using SMEs (*p < 0.5, **p < 0.01).

Liking ratings for the low art-expertise group were analysed using a 2x2 (*phase* x *set*) repeated-measures ANOVA. This revealed a significant main effect for *set*, *F*(1,11) = 11.53, *p* = .0060, η_p_^2^ = .512. Importantly, the effect of *phase* was qualified by a significant interaction between *phase* and *set*, *F*(1,11) = 5.68, *p* = .0363, η_p_^2^ = .341. No other effects were significant. Fig 2 depicts the pattern of results that mirrors the elaboration-based mastering effect found in Experiment 1.

The interaction was further investigated with Bonferoni-adjusted simple main effects of *phase* separately for both sets of portraits. Critically, *mastery* portraits were significantly stronger preferred at t2 compared to t1 (*M*_*diff*_ = .77), *F*(1, 11) = 7.41, *p* = .020, _p_^2^ = .403, while no such effect occurred for *fluency* portraits (*M*_*diff*_ = -.30), *F*(1, 11) = 1.17, *p* = .3040, *ns*, _p_^2^ = .096. Moreover, simple main effects indicated that *fluency* portraits were preferred over *mastery* portraits in t1 (*M*_*Diff*_ = 1.95), *F*(1, 11) = 18.78, *p* = .0012, _p_^2^ = .631, but this difference vanished after repeated evaluation at t2 (*M*_*Diff*_ = .88), *F*(1, 11) = 3.18, *p* = .10, *ns*.

#### Ratings of Liking: Art experienced participants

Likewise, the liking ratings for the high art-expertise group were analysed with a 2x2 (*phase* x *set*) repeated measures ANOVA with no significant effects. Simple main effects for *phase* were carried out separately for both sets of portraits. These results indicated that liking was not significantly affected, neither for *mastery* portraits, (*M*_*Diff*_ = .28), *F*(1, 11) = 2.85, *p* = .1194, *n*.*s*., nor for *fluency* portraits, (*M*_*Diff*_ = -.08), *F*(1, 11) = 3.18, *p* = .6524, *n*.*s*. As in Experiment 1 participants with higher expertise showed no systematic preference effects of repeated evaluation. Simple main effects for *set* revealed that the high expertise group neither preferred *fluency* over *mastery* portraits at t1, *(M*_*Diff*_ = -.22), *F*(1, 11) = .27, *p* = .6157, *n*.*s*., nor at t2, (*M*_*Diff*_ = .15), *F*(1, 11) = .18, *p* = .6765, *n*.*s*. No further effects were significant.

#### Ratings of Atypicality

A 2x2 repeated-measures ANOVA with *phase* (t1, t2) and *set* (mastery, fluency) as independent factors on mean ratings of atypicality revealed a main effect of *set*, *F*(1,11) = 80.08, *p* < .0001, η_p_^2^ = .914, as well as an interaction between both factors, *F*(1,11) = 4.98, *p* < .0474, η_p_^2^ = .312. No other effects were significant. Bonferroni-corrected simple main effects indicated that the interaction was due to an increase in perceived atypicality for *fluency* portraits from t1 (*M* = 2.40) to t2 (*M* = 2.98), *F*(1, 11) = 5.59, *p* = .038, _p_^2^ = .337. Critically, simple main effects confirmed that differences in *atypicality* between *fluency* and *mastery* portraits were highly significant for both phases, t1 (*M*_*diff*_ = 3.00), *F*(1, 11) = 214.08, *p* < .0001, _p_^2^ = .951 and t2 (*M*_*diff*_ = 2.20), *F*(1, 11) = 36.46, *p* < .0001, _p_^2^ = .768. The large effect sizes indicated that despite a slight decrease in contrast ratings of *atypicality* remained remarkably distinct across both phases.

Again, we crosschecked results conjointly for both expertise groups with a mixed 2x2x2 (*phase* x *set* x *expertise*) repeated-measures ANOVA with no additional *expertise* main effects or interactions. The main effect of *set* as well as the interaction between *phase* and *set* remained significant. Therefore, the distinct patterns of preferences between lower and higher art-experts could not be attributed to differently perceived levels of *atypicality*.

#### Summary

Results of Experiment 3 replicated the initial pattern found in Experiment 1 with a novel and larger portrait pool indicating that the underlying preference mechanism is stable and robust. Moreover, this validated the second stimulus pool as suitable for an application in a modified follow-up task of Experiment 4.

### Experiment 4: Repeated evaluation transfer task

In Experiment 4, we applied a modified repeated evaluation task, in which novel but stylistically highly similar portraits of the same artists were shown at test phase (t2). This procedure enabled observing whether the presumed elaboration-based mastering effect that had been established in Experiment 3 would *generalize* across different exemplars of the same artists, or, alternatively, whether the effect would be restricted towards each unique exemplar. This question of object-specificity allowed to further specifying the nature of underlying mental processes for the reported effect. As discussed in the introduction, according to a visual adaptation (perceptual learning) hypothesis, which describes a memorial adaptation-mechanism based on processing of superficial appearance, the observed preference effect should generalize onto new exemplars of the same artist-specific prototypical styles (i.e. to depend on “types”). Alternatively, if the effect would be constrained to the very portrait, this would imply the underlying mental mechanism is associated with processing of embodied semantic information that is unique to each portrait (i.e. to depend on “tokens”). Since constructive “top-down” driven processes are rather idiosyncratic [132], a strong object-dependence would further corroborate the corresponding mechanism’s meaning-driven orientation, in line with our cognitive mastering hypothesis.

#### Subjects

24 participants (23 female) from the University of Vienna participated for partial course credit. Mean age was 22.0 years (range: 18–42 years).

#### Stimuli and apparatus

The same stimuli as in Experiment 3 were used. Again, the apparatus remained the same as described in Experiment 1.

#### Procedure

Compared to Experiment 3 the procedure was modified by a test phase (t2), in which novel portraits of the same styles by the same 10 artists were presented (with five mastery and five fluency portraits). The order in which pairs of portraits were shown (subset A-B vs. B-A) was balanced across subjects to control for sequence-effects and potential differences in perceived artistic quality. In a post-hoc paper and pencil test the degree of similarity between pairs of portraits was assessed from each participant individually. The repeated evaluation phase was identical to Experiment 1 and 3.

### Results and Discussion Experiment 4

First, we ran a mixed 2x2x2 (*phase* x *set* x *expertise*) repeated-measures ANOVA on mean ratings of liking sampled over participants. Since this revealed no main effects or interactions with *expertise* we carried out a 2x2 (*phase* x *set*) repeated-measures ANOVA. This analysis showed significant main effects for *phase*, *F*(1,23) = 4.90, *p* < .0076, η_p_^2^ = .271 and *set*, *F*(1,23) = 19.00, *p* = .0003, η_p_^2^ = .451 as well as a significant interaction between both factors, *F*(1,23) = 80.08, *p* = .0002, η_p_^2^ = .452. As shown in Fig 2 the interaction was due to a drop in ratings of liking for *fluency* portraits, (*M*_*diff*_ = -.94), *F*(1,23) = 27.75, *p* < .0001, η_p_^2^ = .547 that was not observed for *mastery* portraits, for which preferences remained stable across phases (*M*_*diff*_ = .04), *F*(1,23) = .04, *p* = .8385, η_p_^2^ = .002. To rule out a possible confound between effects on preferences for *mastery* portraits and differences in perceived similarity of pairs of portraits between artists, we analysed Spearman-Brown correlations between similarity ratings and changes in liking for *mastery* portraits. This revealed no significant correlation between both variables, *r* = .306, *n* = 24, *p* = .146, *n*.*s*. Furthermore, a t-test indicated no significant difference in similarity ratings between the sets of *fluency* and *mastery* could be found, (M_*Diff*_ = .10), *t*(24) = .47, *p* = .6429, *n*.*s*.

#### Summary

Findings for *mastery* portraits indicated that preferences for target portraits (although resembling the corresponding study portraits very closely) remained at the same baseline level of an initial rating despite the repeated-elaboration phase. Thus, the preference effect obtained in Experiment 3 did not generalize onto novel portraits and was constrained to each specific portrait. This strong object-dependence conflicted with a perceptual learning hypothesis but becomes plausible and was predicted by a cognitive mastering hypothesis. Moreover, the significant preference drop for *fluency* portraits, though unexpected, may contain valuable information: This further corroborated that *fluency* and *mastery* portraits were autonomously susceptible towards the experimental manipulation, in line with a dual-process interpretation (see General Discussion). Although the preference-drop for *fluency* portraits cannot be resolved with certainty, the exposition towards novel portraits at t2 might have caused a sudden disruption of processing ease for per se highly fluent portraits. In other words, due to *fluency* portraits’ high clarity, unambiguity, and realism, they may have been particularly prone to disfluency effects as a result of misleading expectations. Belke, Leder, Strobach and Carbon [30] showed that such disfluency effects (resulting from misleading title primes) were more strongly pronounced for representational as compared to abstract or semi-abstract paintings.

Noteworthy, the reported effect for *fluency* portraits should be carefully interpreted within the confines of an experimentally controlled stimulus-generalization task. Without doubt, being suddenly exposed to a novel portrait (immediately after having processed another exemplar by the same artist) constitutes an artificial viewing situation that does not intend to simulate real-life encounters with art. Therefore, the effect of a strong preference-decrease for *fluency* portraits may hold interpretable information but does not imply that viewing several fluent artworks of the same style could not be enjoyable under other conditions.

## General Discussion

We have outlined that fluency theory predominates current research on human aesthetics [3] and that a fluency-like rationale underlies popular “aesthetic laws” proposed in neuroaesthetics. This, however, leaves largely unexplained what causes mentally challenging or *dis*fluent art experiences to be nevertheless aesthetically rewarding. Though other lines of research cohere at the idea that mental stimulation derived from artistic artefacts is essential to understand why people are attracted them, what seems missing, is a plausible psychological mechanism that explains *how* challenging art gets preferred although it is initially rejected. We have discussed why cognition might be affected by the symbolical/representational status of pictorial art as well as by peculiarities of the art-viewing situation–which both may profoundly shape the formation of aesthetic preferences and, therefore, cannot be simply equated with conditions of every-day viewing situations. Building-up on our model’s assumptions, research on disfluency, as well as long-standing art-philosophical notions on “art as representation” in the tradition of Gombrich [48], we presumed that particularly experiences of challenging art require semantic mastering at the level of “aboutness” in order to be experientially pleasing. Here, we investigated whether such semantic mastering can be achieved through cycles of cognitive elaboration–without offering any “biasing” additional information or provoking intense “aha!” moments–which should meet conditions of a typical art-encountering situation.

As stimulus material, we utilized two types of portraits that differed in their overall degree of mental accessibility. The corresponding dimensional structure was empirically revealed by means of an extensive PCA that preceded the experiments. Results of this PCA demonstrated that portraits (although initially selected based on *atypicality* ratings) systematically differed on multiple perceptual and cognitive dimensions but coherently formed two complementary clusters: One positively associated with high processing-fluency (labelled *fluency* portraits) and one with a high cognitive stimulation potential (labelled *mastery* portraits).

In sum, collective evidence from four experimental tasks confirmed the idea of an aesthetically pleasing elaboration-based mastering mechanism for mentally challenging portraits. In particular, in a repeated evaluation task (Experiment 1), in which portraits were intensively elaborated in respect to numerous evaluation scales, art-inexperienced participants showed a selective increase in liking for mentally challenging portraits from a first to second viewing phase. Critically, this effect was double dissociated: First, it was absent in the visual exposition conditions (i.e. neither emerged in the familiarity condition of Experiment 1 nor in the mere exposure condition of Experiment 2 for art-inexperienced viewers). This ruled out the effect could be traced towards repeated measuring, enhanced mere exposure, visual familiarity, or perceptual fluency, all of which would have constituted passive forms of pre-reflective mental regulation. Secondly, no comparable effect could be found for *fluency* portraits, for which preferences remained remarkably stable across experimental conditions (of Experiment 1–3) and levels of art-expertise. This finding is incompatible with the assumption that elaboration of portraits enhanced aesthetic preferences *per se* (i.e. interpretation by itself) would be pleasurable. Instead, findings supported the idea of a challenge-based response mechanism that selectively operated for non-fluent portraits. As outlined in the introduction, the logic of a mastering mechanism presumes the subjective experience of metacognitive difficulty, that is, the occurrence of excessive mental processing demands resulting from disfluent object-characteristics, in order to exert phenomenally pleasing processing [100, 101]. The robustness of this elaboration-based mastering effect was corroborated in a repeated evaluation replication task (Experiment 3) using a novel and more encompassing pool of portraits, which indicated the effect may be of a general nature. Furthermore, in an explorative repeated evaluation transfer task (Experiment 4) the effect did not generalize onto novel portraits of the same artists, although stylistic similarities between pairs of (study and target) portraits were clearly perceived (and differences in visual similarity between artists did not moderate its strength). This strong dependence on each unique exemplar (“token”) discounted the idea of a perceptual adaptation effect towards artistic-specific visual prototypes. Such perceptual form of learning–based on modified memorial representations linked to atypical category members, see [143, 145]–should have allowed generalizing preferences within “types” of the same prototypical appearance. In other words, had repeated elaboration lead to refined perceptual representations of e.g. a typical Picassoesque manner of representing a human face, this should have applied to several “Picassos” and led to comparable preference rates. Therefore, results of Experiment 4 suggested that mastering activities were oriented on unique expressive semantic content (”aboutness”) rather than formal stylistic appearances, which further indicated its meaning-driven nature.

Conjointly, the results of the study supported our assumption that challenging portraits, in order to be aesthetically pleasing, require deliberate cognitive activity at what has been coined the “reflective system” [162]. This finding that “art requires work” opposes a still popular belief in psychophysical, computational oriented research, and models in Berlyne’s psychobiological tradition to regard aesthetic pleasure primarily as a function of visual perception, see [109]. Instead, the study’s results are in line with current perspectives in evolutionary aesthetics e.g. [1, 61, 163] and long-standing art-philosophical notions that cognition of art is rooted in a special kind of symbolical object understanding that “switches” mental activities towards distilling of represented meaning beyond analysis of superficial appearance (see introduction). This interpretation offers an alternative view of why laboratory induced mere exposure effects did not show-up in this study and were often inconclusive when real art is the object of study [19, 122, 130]. Although different mechanisms for mere exposure effects, such as enhanced familiarity, perceptual fluency, or perceptual uncertainty-reduction explanations have been propagated [19, 121], none of these conceptions of passive implicit mental activities seem to translate well to the mental “affordances” posed by challenging art.

Moreover, aesthetic pleasure derived from elaboration-based mastering appeared to be a time-dynamical process that is neither predicted by fluency theory nor by static aesthetic formulas (e.g. of “optimal arousal” models), and, therefore, would have been obscured by a single measurement. This temporal aspect of aesthetic pleasure has already been acknowledged in Fechner’s “principe” [83] as a hallmark of aesthetic experiences but has often been overlooked in contemporary experimental psychology [99].

We propose to consider the following explanations why elaboration of difficult-to-process portraits became aesthetically pleasurable: In line with established semantic network theories of human memory [164–166] evaluation scales might have spread activation across associative networks and thereby affected a viewer’s phenomenal processing experience. This could be thought of in several ways: Repeated evaluation scales might have primed a wider network of previously unrelated cognitive units and increased perceived meaningfulness, which is widely considered a key-determinant of aesthetic preferences [125, 167]. Alternatively, the contained word labels may have served as cognitive reference points that allowed for a “top-down” directed interpretation that enhanced feelings of phenomenal processing success [78, 151, 152] or conceptual coherence [29]. Moreover, repeated-evaluation may have induced a self-controlled and sustained type of response-based contemplation (see final section of this discussion).

To a various extent, the aforementioned processes imply shaping or extension of pre-existing knowledge, hence the occurrence of learning and mental growth. This idea is sustained, as repeated evaluation is known to prompt deeper processing of target stimuli [156], and, therefore, might foster pleasurable visual learning experiences in the manner proposed by Biederman and Vessel [70]. This would further explain the effect’s selectiveness towards challenging portraits that–due to strong novelty–seem predestined to foster abstract concept formation as described by Watanabe [168]. Although the current study cannot resolve which of these non-mutually exclusive alternatives applied, they exemplify plausible reward-associated mechanisms that are clearly dissociated from immediate fluency (“easy processing is aesthetically pleasing”).

The study’s results are therefore in line with a dual process view of aesthetic pleasure: First, both sets of portraits were associated with distinctive preference patterns. Opposed to *mastery* portraits preferences for *fluency* portraits remained consistently stable across conditions, points of measurement, and levels of art-expertise. This indicated an underlying preference mechanism that neither required extensive elaboration nor prior visual training, in line with the idea of fluency as an early spontaneous positive affect response [12] that serves an implicit processing goal of preservation and conformation of knowledge [16]. Secondly, preferences for *mastery* and *fluency* portraits could, to an extent, be manipulated independently of the respective other set across the experimental conditions (i.e. repeated evaluation selectively affected liking for *mastery* portraits but not for *fluency* portraits–while the reverse pattern was found in the cross-stimulus transfer task). Moreover, art-expertise moderated preferences for both sets differently in the repeated evaluation conditions and the familiarity condition (see below). Such autonomous susceptibility is expectable if preferences were mediated by two distinct mechanisms (one associated with processing-fluency and one with elaboration-based mastering). Previously, a dual-process view for aesthetic preference formation has been propagated by Hekkert and van Wieringen [169], whose comprehensive account integrates several well-established general psychological findings (for a review see [170]). In a similar vein, Armstrong and Detweiler-Bedell [16] proposed to distinguish “between the […] pleasure associated with familiar or easily categorized objects and the exhilaration associated with objects that challenge the mind’s ability to understand them” (p. 306). However, it seems noteworthy that our distinction is compatible though not equateable with the aforementioned accounts. First, our distinction is not that of automatic perceptual versus deliberate cognitively driven processes since processing fluency is known to affect conceptual ease [13, 14, 24, 25]. This was reflected in the PCA results of this study showing that *fluency* portraits (compared to *mastery* portraits) systematically scored higher in several variables assessing semantic accessibility. Thus, both proposed mental mechanisms may encompass cognitive activity, consistent with Kahneman’s [107] dual-process view of intuition-based (“System 1”) and reason-based (“System 2”) mental activities that, accordingly, both transcend implicit, perceptual processing. Further, according to the outlined assumptions on symbolical cognition, perceiving art requires a state of deliberate conscious awareness [116] or specific “cognitive orientation” [49] to translate into meaningful experience–regardless of the level of mental ease an artistic artefact can be processed with. Second, different from previous dual process accounts in general psychology the proposed elaboration-based mastering mechanism is exclusive to perceiving art (i.e. applicable only to artefacts possessing “aboutness”). This is why comparable elaboration effects cannot be expected in every-day viewing situations, e.g. to account for aesthetic preferences of plain objects such as faces or landscapes, which constitute divergent processing demands. However, assumptions may hold for preference formations of designed objects that convey metaphorical content, see [171].

Noteworthy, we are well aware that results reflect perceived differences of inherent variations *relative* to each other’s portrait set. Such a conjoined within-subjects stimulus presentation is known to raise awareness for stimulus-inherent dimensions compared with a homogeneous between-subjects presentation [142]. Therefore, we do not imply that e.g. da Vinci’s “Ginevra de’ Benci” would not possess any mentally stimulating features (resulting from the sitter’s indistinct gaze direction or “sfumato” painterly appearance). However, in direct comparison to e.g. Baselitz’s or Brown’s highly perplexing modes towards representing a human face its fluency characteristics seemed to have clearly prevailed.

### Boundary conditions and future directions

Of main interest for the objective of the study were “pure” effects of art-cognition that could be most properly obtained with participants possessing little prior experience in the visual arts. Therefore, art-expertise specific effects were the study’s main boundary condition. In particular, it appeared that elaboration-based mastering effects were systematically weakened (Experiment 1) or even absent (Experiment 3) when participants possessed of some degree of art-experience (measured by the amount and sophistication of art-specific implicit and explicit knowledge). One tentative explanation for this finding is that increased art-expertise enabled participants for a self-induced mastering of challenging portraits, which had rendered experimentally fostered elaboration redundant. This interpretation is supported by findings of Experiment 1 in which relative art-experienced participants showed systematically increased liking for *mastery* portraits in the familiarity condition although elaboration was not experimentally encouraged. Critically, this effect diminished in the mere exposure condition, in which a stricter time-frame was applied known to prevent deeper cognitive elaboration of paintings [161]. Accordingly, art-experienced viewers can be assumed to already have acquired strategies how to look and evaluate art [109] and to posses refined mastering proficiency [78]. Evidently, such competence should come to full effect only under adequate time-demands (i.e. in the familiarity condition). Moreover, such expertness knowledge and corresponding visual search strategies might have easily interfered with the scales provided in the repeated evaluation conditions. This would mirror findings on conflicting (memorized vs. novel) types of information in human rule-based learning, see [172]. Alternatively, scales might have “trivialized” challenging portraits by constraining potential meanings towards rather obvious characteristics as reported in two previous studies [151, 173]. Both (admittedly speculative) possibilities may exemplify why the repeated evaluation tasks did not enhance appreciation of challenging portraits for more experienced viewers.

Moreover, supplementary regression analysis (not reported in the results section) indicated a kind of step-function separated by a threshold of mid-level art-expertise at which effects of repeated elaboration on appreciation became non-systematic. This pattern and a relatively high intraclass correlation coefficient were indicative of two separate clusters that responded differently to our repeated evaluation manipulations. This corroborated that dichotomizing the sample based on participants’ art-expertise score was reasonable. Art-expertness may be regarded a moderating competence that, once acquired, induces a substantial qualitative change in the processing of challenging art (e.g. in respect to a distinctive search for meaning in art [98, 127, 174, 175]). Alternatively, art-expertness may be associated with higher degrees of specific personality traits (such as openness to experience [176]; sensation seeking [177]; need for cognition [178]; or ambiguity tolerance [179]) thus elaboration effects might have been moderated by dispositional tendencies. However, in the absence of further data these notions have to remain speculative and need to be investigated by subsequent research in more detail.

Furthermore, in line with previous research in aesthetics e.g. [132, 169, 180, 181] we utilized portraits to address fundamentals in art perception. One may therefore question the ecological validity of the study’s findings. However, this approach becomes plausible in light of recent conceptions of processing fluency [8, 13, 182] as well as our model’s predictions on cognitive mastering [78, 139] that both describe the corresponding mechanisms as ubiquitous phenomena that can be derived in metacognitive manner from a wide array of determinants. This is why fluency predictions are usually neither differentiated for object-domains [7] nor even distinguished between art and non-art [4]. Similarly, our model predicts outcomes of its processing stages to depend on specific key-determinants regardless of the exact type of aesthetic information that feeds into its stages (since any two artworks may vary in an indefinite number of aspects on a “retinal level”). Consequently, although the stimulus selection was clearly restricted, we propose that similar findings (at least in principle) should apply to a broader range of artistic artefacts despite potential variations in central stimulus-dimensions (such as subject matter, colorization, or image size) unless they *alter* the experience of processing ease or mental challengingness. However, future research is needed to confirm this assumption or to reveal potential third-variable boundary conditions.

A further point of debate may be the lack of a preference-generalization effect in Experiment 4 as a seemingly counterintuitive result. Importantly, the repeated evaluation scales of this study referred in Panofsky’s [183] terms to a “pre-iconographical” level of art-interpretation, defined by an analysis of apparent properties that are intrinsic to a particular painting. Accordingly, repeated evaluation did not foster so-called “iconographic” or “iconological” levels of interpretation that would have involved in-depth knowledge of specific themes, universal ideas, or cultural specific contextualization. This further explains why mastering effects were restricted towards particular exemplars as only the latter two levels encompass generalizable forms of knowledge that can be expected to translate onto similar works (e.g. of the same artists, historical period, or subject matter).

From a methodological point of view, to avoid fatigue effects, a repeated evaluation task necessitates to constrain the number of stimuli as reflected in the design of two predecessor studies [140, 141]. Therefore, a potential concern may be that remembering initial rating scores could have biased participants’ responses during test towards consistency. However, this would have yielded in rather static rating-responses, which is incompatible with the data showing selective dynamical changes across multiple conditions. Moreover, task demands of the extensive evaluation/viewing blocks, which encompassed 200 trials, made unrequested recall of each of the initial rating-scores (i.e. atypicality, liking, realism, and complexity) presumably difficult. For a full control of memory-based carry-over effects future research may replicate findings with a between-subjects repeated evaluation paradigm.

Finally, a strict fluency advocate may argue that the proposed cognitive mastering mechanism is tantamount with disfluency-reduction, as both phenomena appear to be closely interlinked. Such interpretation would be in line with Graf and Landwehr’s [184] recent fluency-based model that also presumes a dual-process of two fundamentally distinct information-processing styles in human aesthetics (i.e. one immediate fluency-based and one elaborate perceiver-driven process). This consensus aside, a strict fluency oriented explanation of the latter process is challenged by the current study in two aspects: First, a disfluency-reduction interpretation conflicts with the robust finding that perceived portrait atypicality remained unaffected by elaboration. More particularly, results of the repeated evaluation tasks (of Experiment 1 and 3) demonstrated a clear dissociation between preference and atypicality ratings: While elaboration increased preferences for *mastery* portraits it revealed no systematic effect for their atypicality ratings, which persisted to be very high at t2. Since object-typicality is widely regarded as an indicator for the processing-fluency of an object category e.g. [4, 185, 186], the pattern of results is not in accordance with a disfluency reduction hypothesis. This would have predicted, preference increases and disfluency reduction (as indicated via atypicality ratings) to be correlated. Noteworthy, results mirrored findings of preferences for atypical (disfluent) designed artefacts under elaboration e.g. [142, 157], which rules out to be regarded as a mere artefact of this study. Therefore, results hold evidence that preference formation for disfluent aesthetic artefacts can occur without changes in its associated processing fluency. This interpretation is in line with a cognitive mastering mechanism that we have primarily linked to the pleasure of mental growth and vision-based conceptual learning. Consequently, a mastering mechanism amounts to fluency-unrelated predictions. To give just one example, elaboration of disfluent art might tap into pleasure of knowledge attainment by offering the prospect thereof irrespective of its actual completion, see [16].

A second concern against a disfluency-reduction interpretation regards the nature of such effect as proposed in fluency theory. Therein, processing of challenging modern art is essentially described as a kind of problem-solving behaviour that necessitates full cognitive resolution (“having solved the puzzle”) in order to translate into aesthetic pleasure [4, 187]. Therefore, disfluency-reduction directly lends itself to account for Aha-experiences [43, 57] or cognitively controlled disambiguation effects in aesthetics [30]. However, the design of the repeated evaluation task makes the occurrence of such effect improbable. Specifically, the provided repeated evaluation scales neither contained disambiguating cues (e.g. semantic information that had eased finding unapparent conclusions or perceptual Gestalts) nor offered portrait specific information. Considering as well the time characteristics of the repeated evaluation procedure (that lasted 40 minutes), these are task demands not known to foster sudden fluency, but, instead, seem to establish a sustained type of aesthetic reflection and contemplation that evolves over time.

The above considerations mark the key difference between a disfluency-reduction and elaboration-based mastering conception on a theoretical level: While the explanans for reward in fluency theory is the outcome or processing-*endpoint* of an aesthetic episode, a mastering hypothesis presumes that information processing-*dynamics* as such (i.e. the “liveliness” of higher-order mental faculties at work) to be reward-associated. The latter seems better suited to cover the pattern of results and to account for the task demands applied in the repeated evaluation conditions. Accordingly, repeated evaluation–if challenged by disfluency characteristics and directed by specific criteria “how to look at the portraits” (such as the applied ratings)–could be thought of as dynamical interplay of ongoing cognitive contemplation and cycles of re-interpretation as an enjoyable experience itself. This idea is implied in the model of aesthetic experiences [78] by the iterative flow of information processing at its higher-order processing stages. In a similar vein, Lehne and Koelsch [188] have recently proposed an emotion model of tension and suspense, in which aesthetic pleasure follows from future-directed mental processing (i.e. constantly modulated cycles of fulfilling and violating expectations) that can span large time intervals.

To conclude, the final section of this General Discussion pointed at several differences for the theoretical underpinning of how aesthetic preferences for challenging art could be formed. The current study provides evidence for an elaboration-based mastering mechanism as a self-contained and coherent explanation for reward causation from processing disfluent portraits. Since further knowledge is currently not available, future research is needed to refine a dual-process view in aesthetics further.

## Supporting Information

S1 Fig Overview of portrait sets and corresponding artists applied in Experiment 1–3. (TIF)

S2 Fig Overview of *fluency* portraits and corresponding artists applied in Experiment 4&5. (TIF)

S3 Fig Overview of *mastery* portraits and corresponding artists applied in Experiment 4&5. (TIF)

S1 Text Rating Study and PCA Analysis. (DOCX)

S2 Text List of ratings utilized in the repeated evaluation phase of Experiment 1 and 3. (DOCX)
