# Correction: When Challenging Art Gets Liked: Evidences for a Dual Preference Formation Process for Fluent and Non-Fluent Portraits

**DOI:** 10.1371/journal.pone.0138962

**Published:** 2015-09-18

**Authors:** Benno Belke, Helmut Leder, Claus-Christian Carbon

The third author’s name is incorrectly spelled. The correct name is: Claus-Christian Carbon. The correct citation is: Belke B, Leder H, Carbon C-C (2015) When Challenging Art Gets Liked: Evidences for a Dual Preference Formation Process for Fluent and Non-Fluent Portraits. PLoS ONE 10(8): e0131796. doi:10.1371/journal.pone.0131796
