# Supplementary figures and images for: When Challenging Art Gets Liked: Evidences for a Dual Preference Formation Process for Fluent and Non-Fluent Portraits
Source: PLoS One. 2015 Aug 26;10(8):e0131796. doi: 10.1371/journal.pone.0131796 (PMC4550383; doi:10.1371/journal.pone.0131796)

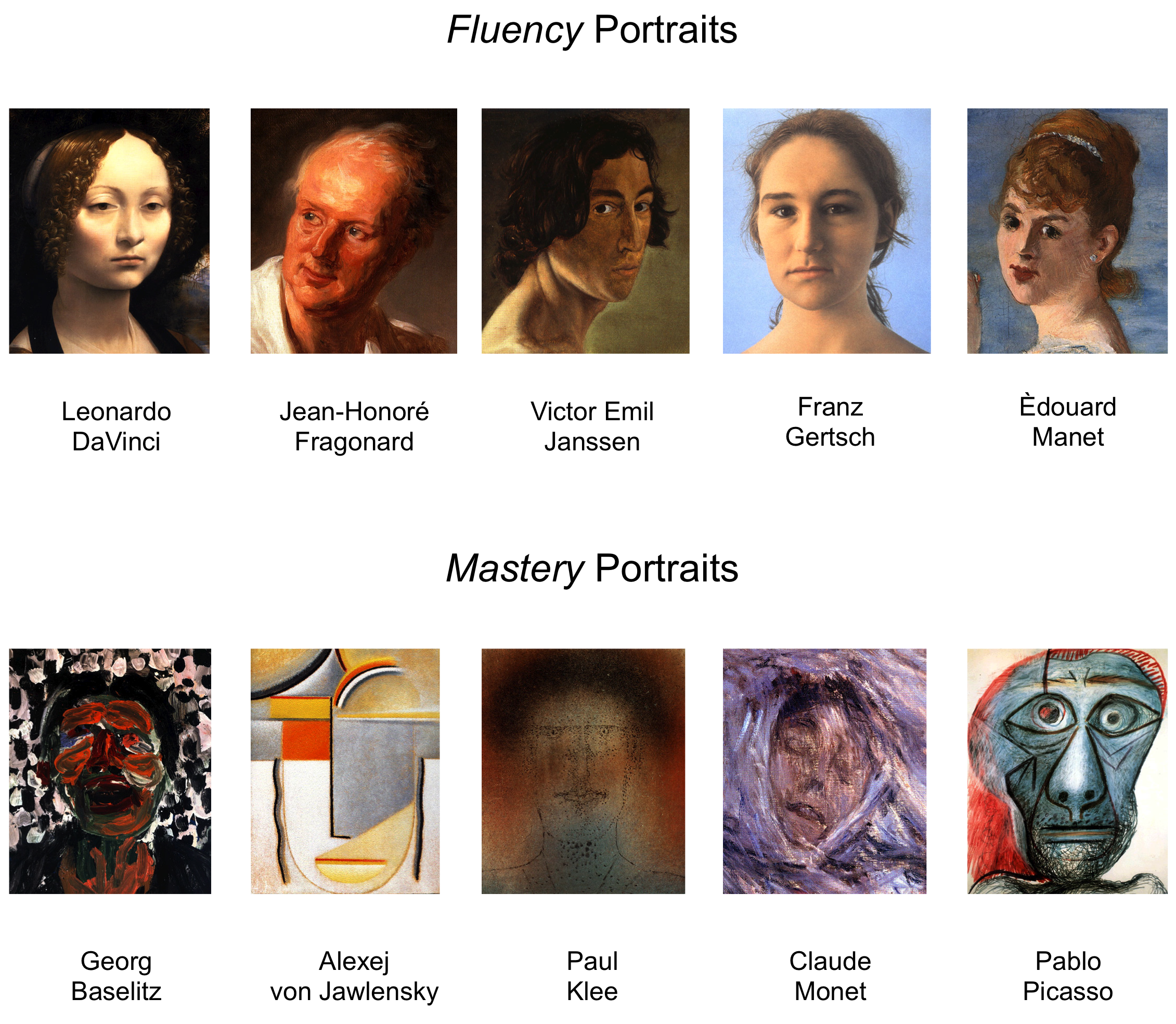

Supplement: S1 Fig — (TIF) [file pone.0131796.s001.tif]

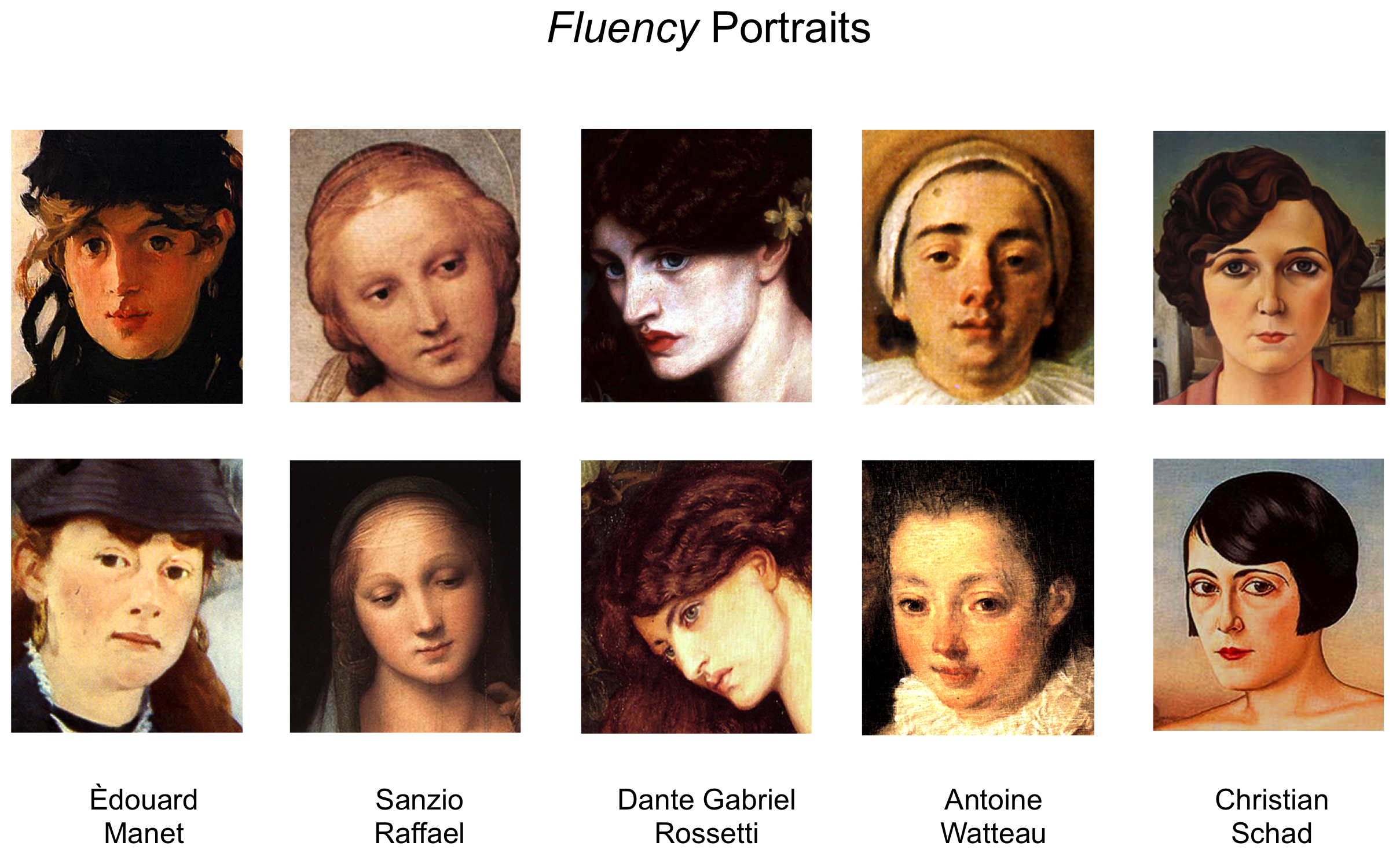

Supplement: S2 Fig — (TIF) [file pone.0131796.s002.tif]

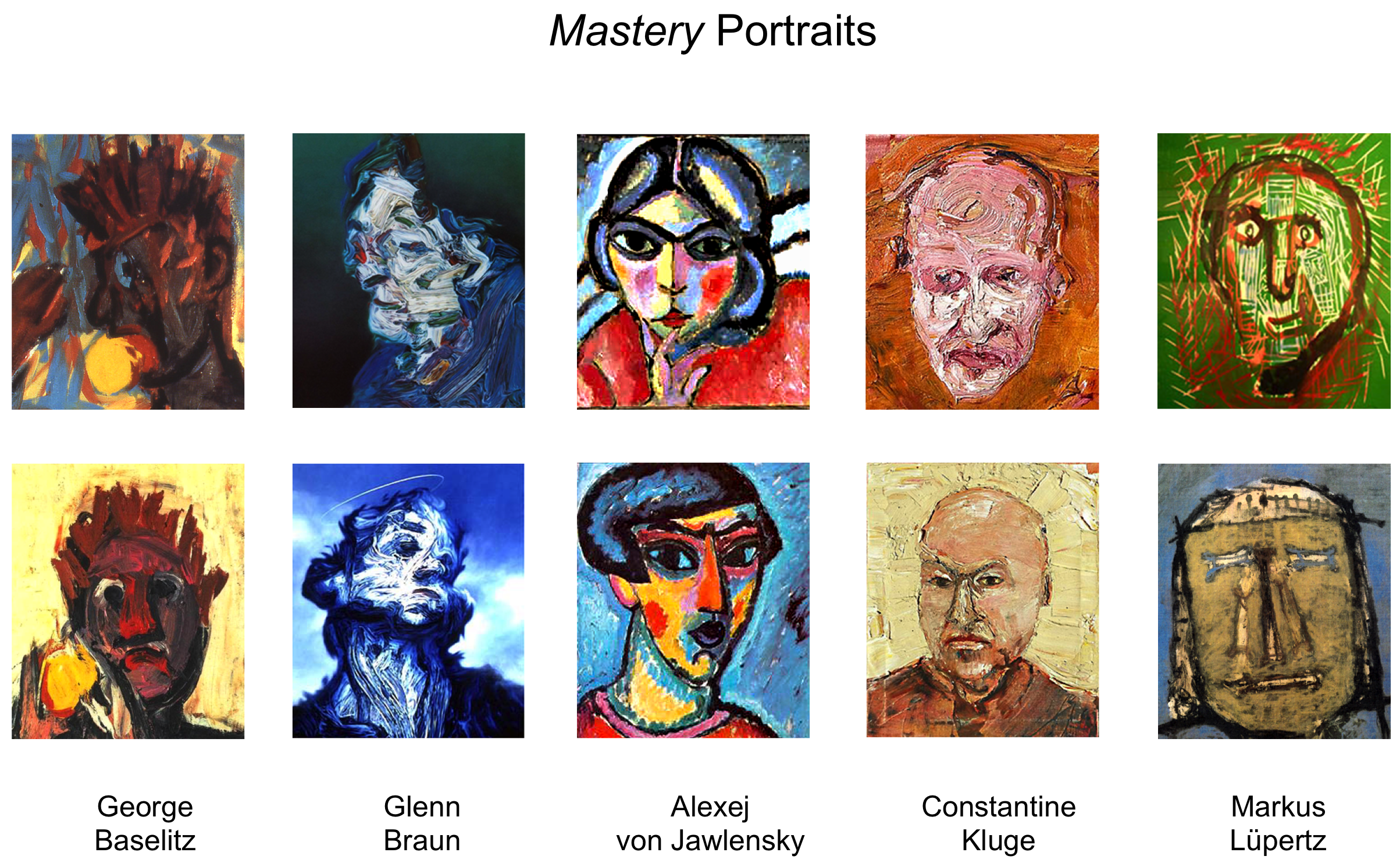

Supplement: S3 Fig — (TIF) [file pone.0131796.s003.tif]
