# Supplementary material for: When Challenging Art Gets Liked: Evidences for a Dual Preference Formation Process for Fluent and Non-Fluent Portraits
Source: PLoS One. 2015 Aug 26;10(8):e0131796. doi: 10.1371/journal.pone.0131796 (PMC4550383; doi:10.1371/journal.pone.0131796)
Supplement: S1 Text — (DOCX) [file pone.0131796.s004.docx]

**Supporting Information S4. Rating Study and PCA Analysis.**

Here, we provide additional information on the rating study and results of the Principal Component Analysis (PCA).

*Subjects*

Twenty-seven psychology students from the University of Vienna were recruited. The mean age of participants was *M* =26.2 years (range: 24-31 years).

*Apparatus and stimuli*

All 30 portraits of stimulus pool 1 and 2, which were used in the experimental tasks. Two types of scales were applied to obtain the rating-responses. First, participants were asked to rate their endorsement to 22 statements on 9-point Likert response scales (anchored by the poles ‘this statement applies not at all’ and ‘this statement applies completely’) and secondly, according to 14 semantic differential scales, measured by 7-point Likert response scales (using bipolar adjective scales). All items were provided in German language.

*Procedure*

As a paper and pencil test the questionnaire was applied in a group session. Each participant had to rate in total 30 portraits according to 36 items, resulting in 1080 responses per person. Portraits were presented consecutively via overhead projector for four minutes each.

Results & Discussion

To explore the underlying factor structure of the portraits we performed a PCA based on a person-by-object matrix (with persons *and* objects per column). This “three-modal” approach to factor analysis considers three sources of variation conjointly (i.e. subjects, objects, and ratings) and therefore maintains information resulting from person-specific variance, see [1-3], which would have otherwise been suppressed in an aggregated matrix (with just objects or just persons per column). Particularly, the rationale for a three-modal PCA were the following: Due to the nature of impression-formation and self-referential response items of the questionnaire as well as systematic differences between our stimuli that were expected to be reflected in the data, *both* person- and object-related variances were of particular importance for revealing the underlying dimensions. This is specifically guaranteed in three-modal factor analysis [4]. The corresponding correlation matrix revealed that 3 items did not meet the Kaiser-Meyer-Olkin criterion (MSA) of ≥ 0.5 and were therefore omitted from further analyses.

The correlation matrix for the reduced item set revealed an overall MSA value of .931. This corresponds to a marvellous adequacy according to Kaiser, Rice, & Mark´s (1974) classification scheme (for MSA values ≥ .9) and confirmed the suitability of the data for the PCA. For the number of extracted factors we chose the Kaiser extraction criterion with Varimax rotation. After rotation converged in 11 iterations this resulted in extraction of 7 factors with eigenvalues greater one, explaining 67% of the total variance. The eigenvalues of the first two factors put together were 15.2, explaining 46% of variance of the data alone. Table 1 gives an overview of the final set of items and corresponding factors.

In particular, 12 items were related with the first factor, with the marker item *comprehensibility* (factor loading = -.70) and the items *unambiguity*, *attractiveness*, *order*, *realism*, *abstraction*, *determinacy,* *emotional clarity,* *typicality*, *roundness*, *liking* and *autonomy*. This factor was labelled *accessibility*. It subsumed fluency-related qualities on a perceptual as well as conceptual level. The second factor had the marker item *idiosyncrasy* (factor loading = .82), and was related to the items *expressiveness*, *intentionality*, *imagination*, *interest, innovativeness, importance of style* and *atypicality.* This factor was labelled *cognitive stimulation* and mirrored a likewise labelled factor in a recent art reception survey [5]*.* It referred to challenging and mentally exciting qualities similar to Berlyne’s idea of an arousal potential. A third factor, labelled *zeitgeist*, consisted of the two items *aesthetic norms* as the marker item (factor loading = .83) and *conventions.* It characterizes the degree to which aesthetic traditions are traceable in the portraits. The fourth factor, representing *forms* as the marker item (factor loading = .80), as well as *lines*, and *colours* was labelled *stylistic components*. This factor subsumes information on salient surface features of artist-specific styles. Three items were associated with the fifth factor, with *tone* as the marker item (factor loading = .72), as well as *hue* and *mood.* We labelled this factor *affective valence* that refers to an indistinct affective quality of the portraits. The sixth factor represented *coping* as the marker item (factor loading = .82), *meaningfulness*, as well as *familiarity*. It was labelled *coping* as it was best explained with a self-assessed competence to successfully assimilate or accommodate portraits on a semantical level. The last factor, labelled *complexity*, was related to *richness of detail* and *simplicity* and carries information on the overall visual variety and quantity of features.

Noteworthy, inter-correlations between the two main factors were considerably high: *Cognitive stimulation* was negatively associated with *accessibility* (-.59) and with *coping* (-.46). This could be interpreted towards a second order factor structure, in which a general (meta-) factor of “mental challengingness” was anchored by the two poles *accessibility* (low) and *cognitive stimulation* (high). However, the 7-factor structure appeared superior as it complied with results of a previous rating study [5] and contained more fine-grained information.

We further tested, whether our conjoint analysis of all 30 portraits in one PCA was appropriate by testing for potential differences between both portraits pools (i.e. 10 portraits of the first pool compared to the 20 portraits of the second pool). A 2x7 repeated-measures ANOVA (aggregated per person) with *portrait pool* (pool 1, pool 2) and *PCA dimension* (all seven PCA factors operationalized by scores of the corresponding marker items) as independent factors on mean ratings as the dependent variable was performed (see Table 1 for a list of all marker items). This revealed a main effect of *PCA dimension*, *F*(6,21) = 41.67, *p* < .0001, *η*_p_^2^ = .923, resulting from significant differences between nearly all 42 individual comparisons of dimensions. Importantly, results revealed neither a significant main effect of *portrait pool*, *F*(1,26) = .07, *p* = . 7917, *n.s.* nor a significant interaction between the factors, *F*(6,21) = .97, *p* = .4712, *ns*, *η*_p_^2^ = .216. Thus, the portrait pools were not marked by significant differences concerning the PCA factors, which confirmed a conjoint factor analysis was adequate.

Moreover, we tested on which of the seven identified PCA dimensions the two subsets of 15 *mastery* and 15 *fluency* portraits systematically differed. Therefore, we performed a 2x7 repeated-measures ANOVA (aggregated per person) with *set* (mastery, fluency) and *PCA dimension* (all seven PCA factors) as independent factors on ratings of corresponding marker items. This revealed a significant main effect of *set, F*(1,26) = 47.90, *p* < .0001, *η*_p_^2^ = .648, a significant main effect of *PCA dimension*, *F*(6,21) = 41.45, *p* < .0001, *η*_p_^2^ = .922, as well as a significant interaction between both factors, *F*(6,21) = 41.67, *p* < .0001, *η*_p_^2^ = .855. Table 1 shows mean ratings for each marker item separately for both sets. Simple main effects revealed that the interaction was due to differences between *mastery* and *fluency* portraits for *accessibility*, *cognitive stimulation*, *zeitgeist*, *stylistic components*, and *affective valence*, on a *p* < . 0001 significance level. No other differences between the sets reached significance (*coping, p* = .0975, *n.s.*, and *complexity, p =* .2131, *n.s).* Most important, both portrait sets showed a strict complementary factorial pattern: *fluency* portraits were marked by high degrees of *accessibility* and low degrees of *cognitive stimulation*, while *mastery* portraits showed the inverse pattern. The factor structure thereby revealed that the two sets formed two coherent clusters of two hedonic potentials (one associated with easy-processing and one with mental challengingness). Noteworthy, the item *atypicality*, which served as the sets’ initial selection criterion, was positively associated with *cognitive stimulation* (.514) and almost equally high, but negatively with *accessibility* (-.488). Thus, *atypicality* had a high discriminatory power to distinguish between both main factors and proofed a reliable indicator for a spectrum of differences on a perceptual as well as conceptual level.

The finding that *fluency* portraits were also perceived to more strongly express *Zeitgeist*, to be less marked by *stylistic features*, and to have a more positive *affective tone* indicated that perception of paintings were affected by further factors (beyond the two main factors)*.* However, these accounted for only 13.86 % of the total variance. Moreover, *Liking* did not load high on any of these factors compared to *accessibility* (.569) and *cognitive stimulation* (.344). Therefore, potential effects on (and confounds with) preferences resulting from these other factors seemed to be rather marginal.

*References*

1. Bortz J (2005) Statistik für Human- und Sozialwissenschaftler. 6. Auflage. [Statistics for human and social sciences. 6^th^ edition]. Heidelberg: Springer Medizin Verlag.
2. Krolak-Schwerdt S (1991) Modelle der dreimodalen Faktorenanalyse: formale Eigenschaften, theoretische Zusammenhänge und ihre Implikationen für das Konzept individueller Differenzen [Three-mode models of factor analyses: Formal properties, theoretical relations, and implications for the concept of individual differences]. Psychol Beitr 133: 314-346.
3. Tucker LR (1966) Some mathematical notes on three mode factor analysis. Psychometrika 31: 279-311.
4. Backhaus K, Erichson B, Plinke W and Weiber R (2011) Multivariate Analysemethoden [Methods of Multivariate Analyses]. Berlin: Springer.
5. Hager M, Hagemann D, Danner D and Schankin A (2012) Assessing Aesthetic Appreciation of Visual Artworks-The Construction of the Art Reception Survey (ARS). Psychol Aesthet Crea 6: 320-333.
