# Supplementary material for: When Challenging Art Gets Liked: Evidences for a Dual Preference Formation Process for Fluent and Non-Fluent Portraits
Source: PLoS One. 2015 Aug 26;10(8):e0131796. doi: 10.1371/journal.pone.0131796 (PMC4550383; doi:10.1371/journal.pone.0131796)
Supplement: S2 Text — (DOCX) [file pone.0131796.s005.docx]

**Appendix S5. List of ratings utilized in the repeated evaluation phase of Experiment 1 and 3.**

1. How interesting is this portrait?
2. How conservative is this portrait?
3. How expressive is this portrait?
4. How artistically precious is this portrait?
5. How imaginative is this portrait?
6. How distinguished is this portrait in painterly quality?
7. How trivial is this portrait?
8. How inspiring is this portrait?
9. How provocative is this portrait?
10. How kitschy is this portrait?
11. This portrait has symbolical meaning.
12. This portrait is characterised by specific lighting conditions.
13. This portrait’s painterly appearance contributes to the portrait’s meaning.
14. This portrait is thought provoking.
15. This portrait contains a deeper meaning.
16. This portrait is defined by an alienated mode of representation.
17. This portrait is worth a second look.
18. This portrait has an interesting colouring.
19. This portrait’s style is particularly salient.
20. This portrait contains a statement of the artist.
